# Point mutation I634A in the glucocorticoid receptor causes embryonic lethality by reduced ligand binding

**DOI:** 10.1016/j.jbc.2022.101574

**Published:** 2022-01-08

**Authors:** Steven Timmermans, Nicolette J.D. Verhoog, Kelly Van Looveren, Sylviane Dewaele, Tino Hochepied, Melanie Eggermont, Barbara Gilbert, Anne Boerema-de Munck, Tineke Vanderhaeghen, Joke Vanden Berghe, Natalia Garcia Gonzalez, Jolien Vandewalle, Yehudi Bloch, Mathias Provost, Savvas N. Savvides, Karolien De Bosscher, Wim Declercq, Robbert J. Rottier, Ann Louw, Claude Libert

**Affiliations:** 1 VIB Center for Inflammation Research, VIB, Ghent, Belgium; 2 Department of Biomedical Molecular Biology, Ghent University, Ghent, Belgium; 3 Department of Biochemistry, Stellenbosch University, Stellenbosch, South Africa; 4 Department of Pediatric Surgery, Erasmus Medical Center, Sophia Children's Hospital, Rotterdam, The Netherlands; 5 Department of Cell Biology, Erasmus Medical Center, Rotterdam, The Netherlands; 6 Department of Biochemistry and Microbiology, Ghent University, Ghent, Belgium; 7 Department of Biochemistry, Ghent University, Ghent, Belgium; 8 Receptor Research Laboratories, Nuclear Receptor Lab, Medical Biotechnology Center, VIB, Ghent, Belgium

**Keywords:** glucocorticoid receptor, mutational study, A465T, I634A, dimerization, ligand binding, DBD, DNA-binding domain, Dex, dexamethasone, DMEM, Dulbecco’s modified Eagle’s medium, GCR, GC resistance, GR, glucocorticoid receptor, GRE, GC response element, H&E, hematoxylin and eosin, LBD, ligand-binding domain, MEF, murine embryonic fibroblast, PLA, proximity ligation assay

## Abstract

The glucocorticoid (GC) receptor (GR) is essential for normal development and in the initiation of inflammation. Healthy GR^dim/dim^ mice with reduced dimerization propensity due to a point mutation (A465T) at the dimer interface of the GR DNA-binding domain (DBD) (here GR^D/D^) have previously helped to define the functions of GR monomers and dimers. Since GR^D/D^ retains residual dimerization capacity, here we generated the dimer-nullifying double mutant GR^D+L/D+L^ mice, featuring an additional mutation (I634A) in the ligand-binding domain (LBD) of GR. These mice are perinatally lethal, as are GR^L/L^ mice (these mice have the I634A mutation but not the A465T mutation), displaying improper lung and skin formation. Using embryonic fibroblasts, high and low doses of dexamethasone (Dex), nuclear translocation assays, RNAseq, dimerization assays, and ligand-binding assays (and *K*_d_ values), we found that the lethal phenotype in these mice is due to insufficient ligand binding. These data suggest there is some correlation between GR dimerization potential and ligand affinity. We conclude that even a mutation as subtle as I634A, at a position not directly involved in ligand interactions *sensu stricto*, can still influence ligand binding and have a lethal outcome.

Glucocorticoids (GCs) as well as their receptor (GR) are essential for life in mammals. The GR is involved in developmental processes, in metabolism, circadian rhythm, and regulation of the immune response (1). Full GR^−/−^ mice are not viable, and the number of GR mutations found in the human population is very low (2). GR^−/−^ mice die immediately after birth due to respiratory failure, caused by underdeveloped lungs, and they display defects in the heart (3), pancreas (4) and skin (5). The GR is a transcription factor (TF) that belongs to the superfamily of nuclear receptors. It is 777 amino acids (AAs) in humans and 783 AAs in mice. It contains an amino-terminal domain (NTD), a DNA-binding domain (DBD), a hinge region, and a C-terminal ligand-binding domain (LBD) (6). The DBD contains two conserved zinc finger elements (each containing four cysteines), of which the most N-terminal one is responsible for the recognition of a DNA motif and the second one for GR homodimerization. Crystal structures were obtained of the DBD (pdb 1GLU) (7) and the LBD (pdb 1M2Z) (8), but not of the entire GR protein. The DBD crystals allowed defining a region of five AAs, the D-loop, which is part of the second zinc-finger and is important for GR homodimerization (Fig. 1*A*). In the absence of ligand, GR resides in the cytoplasm as part of a protein complex containing several chaperones. Binding of GCs leads to structural changes (9) and results in translocation of GR to the nucleus, where it can regulate transcription through binding to DNA and/or to other TFs, as a GR dimer or a monomer.

**Figure 1:**
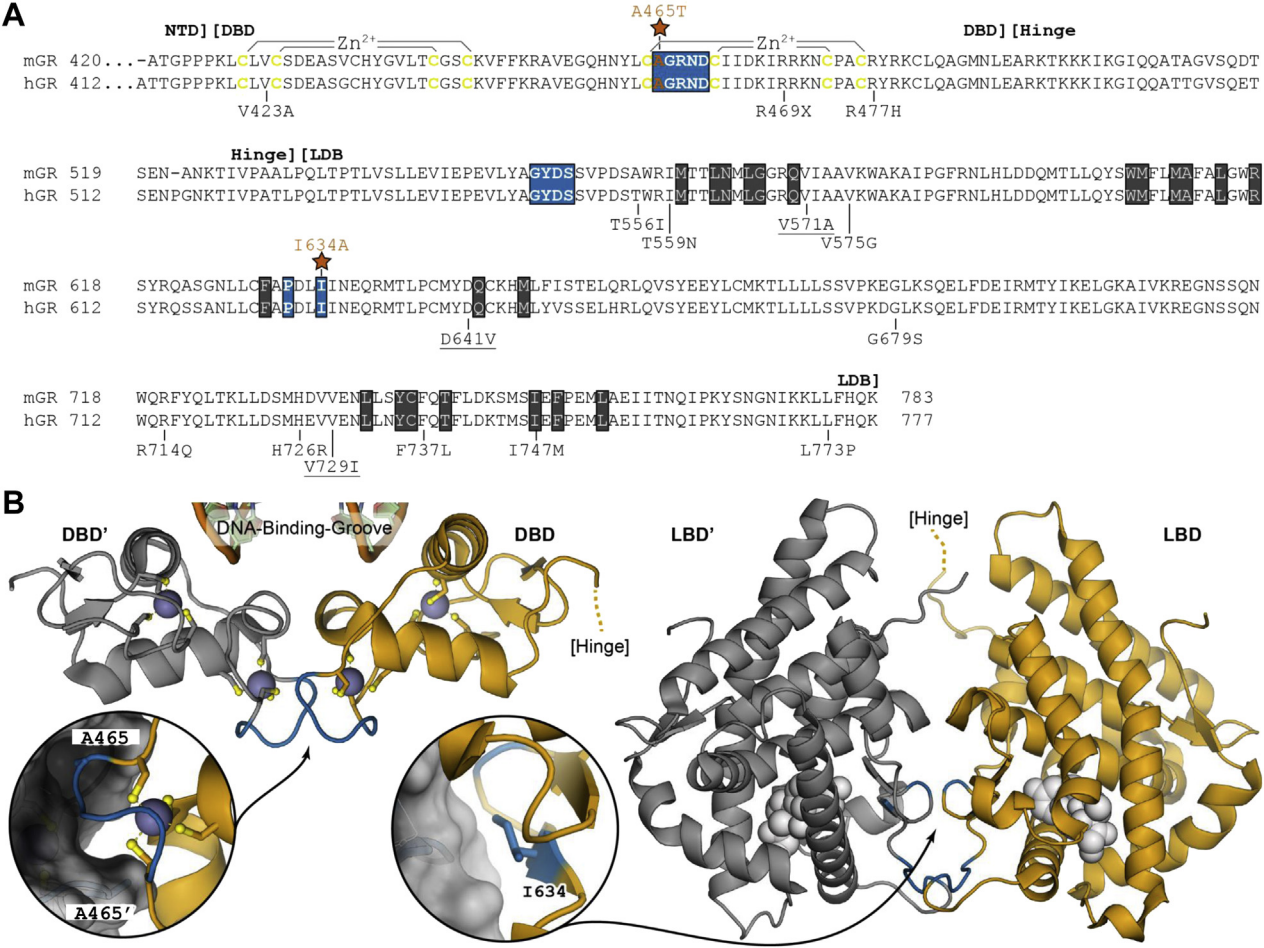
**Overview of the C-terminal GR region and amino acids of interest for this study.***A*, sequence alignment of the C-terminal regions of mouse GR (Uniprot P06537) and human GR (Uniprot P04150) starting from the end of the N-Terminal Domain (NTD) followed by the DNA-Binding Domain (DBD), hinge region, and Ligand-Binding Domain (LBD). In *yellow*, the Cys residues involved in the coordination of the Zinc ions in the two Zinc-fingers of the DBD. Highlighted in *blue* are the homodimerization interfaces of the DBD (D-loop) and LBD. Highlighted in *black* are residues contributing to ligand binding. Marked with a *red star* is the A465 residue, which is mutated in the GR^D^ mice, and the I634 residue, which is mutated in the GR^L^ mice. Pathologic mutations reported by Kino (46) are annotated below the sequences, underlined are mutations that were identified as homozygotic mutations, all others were heterozygotic. Residues involved in ligand binding were retrieved from (8). *B*, *left*, cartoon representation of the dimerized hGR DBD (from pdb 5e69). In *blue*, the dimerization interface as indicated in the sequence. Zinc atoms are displayed as spheres and the coordinating cysteine residues are shown as *sticks*. The *inset* shows a close-up view around residue A465 whereby the alanine side chain of each GR copy occupies a small pocket on the opposing GR. *Right*, cartoon representation of the dimerized GR LBD (from pdb 1m2z). In *blue*, the dimerization interface as indicated in the sequence. The ligand (dexamethasone), near the dimerization interface, is depicted in white spheres. The *inset* shows a close-up view around residue I634 with the side chain conforming to the shape of the opposing GR copy *via* van der Waals contacts. GR, glucocorticoid receptor.

The direct interaction of GR dimers with DNA occurs at a sequence motif called a GC response element (GRE), which is a 15 base pair (bp) motif that is composed of two imperfect hexameric inverted palindromic repeats separated by a 3 bp spacer, with the consensus sequence of AGAACA[N]_3_TGTTCT (10). The spacer allows for two GR molecules (one per hexamer) to bind the DNA in a head-to-head fashion. This stabilizes the interactions with the DNA and between both GR molecules promoting the formation of GR dimers *via* hydrogen bonds (at the level of their DBD) and creating positive cooperativity (11, 12, 13). In addition to this generally accepted dimeric model, there is some evidence, from old structural studies and recent work by Presman *et al.* that higher-order modes of GR interaction may occur in cells and that GR may bind to the DNA as a tetramer (14, 15).

Genome-wide binding studies have shown that only a fraction of all GR-binding sites (GBS) contain a recognizable GRE consensus sequence and GR may bind to very degenerate motifs or may bind indirectly to DNA (16). In addition, there is a second class of binding motifs for the GR comprising inverted repeats (IR-nGRE) with a variable spacer and with a consensus sequence of CTCC[N]_0-2_GGAGA (17). When binding such IR-nGRE sites, the two GR molecules interact in a head-to-tail orientation whereby they show a negative cooperativity (18) and this configuration causes transcriptional repression rather than activation. Furthermore, GR can bind as a monomer to GRE half-site motifs (19, 20). Experimental evidence for such monomeric binding was obtained in mice by exo-ChIP analysis. It was shown that under endogenous (low) corticosterone levels, GR binding to these half-sites is dominant and that in response to exogenous GCs (high levels), GR dimers assemble on full GREs near known genes, at the expense of monomer GR-DNA binding (20).

Synthetic GCs, such as dexamethasone (Dex), are among the most prescribed drugs in the world because of their potent anti-inflammatory and immunosuppressive effects (21). They are used for a wide variety of inflammatory diseases (22), but the optimal use of GC therapy is limited by the occurrence of severe side effects with chronic use (23). Furthermore, there is also a fraction of patients that do not respond to GC-based therapy, a phenomenon that is referred to as GC resistance (GCR) (24, 25). Both problems have led to a search for next-generation GCs, which may have a better therapeutic effect or reduced side effect profile. Structure–function studies of GR have been essential in this search (26, 27). Importantly, several studies have shown that the anti-inflammatory effects of GR are depending on both GR monomeric and dimeric activities, but that the protective effects of GCs in acute inflammation, such as endotoxemia, mainly depend on GR dimer formation. Several GR dimer-induced genes coding for anti-inflammatory proteins have been identified, for example, *Tsc22d3* and *Dusp1* (28, 29, 30, 31). These findings are largely based on studies using a mouse mutant, referred to as the GR^dim/dim^ mouse, officially named Nr3c1^tm3Gsc^, and here referred to as GR^D/D^. The A465T GR^D/D^ mutation targets the alanine found in the D-loop (Fig. 1*A*) and compromises the hydrogen bond formation between two GR monomers, thereby disrupting stable dimerization and positive cooperativity at the level of the DBD (32, 33). In these otherwise healthy GR^D/D^ mice, altered GR activity was shown in a gene- and tissue-specific way (34, 35). Although initially considered to be totally defective in dimerization, some follow-up studies have described that the GR^D/D^ mutation still forms dimers (35, 36), while others suggest that these dimers display less stability and substantially lower dimerization potential (37, 38).

Based on the crystal structure of the LBD (pdb 1M2Z) (8), a second GR dimer interface mediating binding between two LBD moieties of the receptors was identified. Six amino acids appeared crucial for GR dimerization, one of them being isoleucine-634 in mouse GR (Fig. 1*B*). The I634 of 1 GR LBD interacts with P631 of the other GR partner LBD and forms the hydrophobic core of the LBD dimerization domain, which is further stabilized by hydrogen bonds from surrounding amino acids (8). Presman *et al.* (36) have generated cells expressing GR containing the A > T mutation in DBD as well as an I > A mutation in the LBD. These double point mutant GR molecules, called GR^mon^ by the authors, were found to be severely compromised in their GR dimerization potential; however, the *in vivo* consequence of such a double mutation was not yet known.

Aiming to study the relative contribution of the DBD and LBD to GR dimerization interfaces in mice, we decided to generate an *in vivo*, nonfunctional mutation of the I634. Starting from the already generated GR^D^ mutation, we have generated mice comprising also the LBD mutation, leading to GR^D+L^ molecules as well as the single point mutant versions GR^D^ and GR^L^. We found that GR^D/D^ mice were viable, but GR^L/L^ or GR^D+L/D+L^ mice were not. Our results suggest that the mechanism leading to the perinatal lethality of mice carrying the GR^L/L^ or GR^D+L/D+L^ mutation is caused by reduced ligand binding.

## Results

### Generation of GR^L^ and GR^D+L^ mice

**Figure 2:**
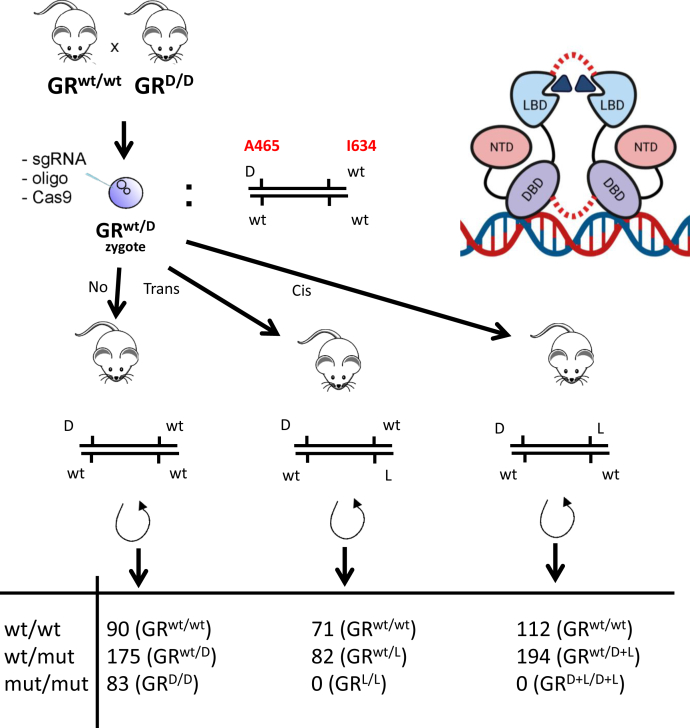
**Generation of GR point mutant mice and study of their phenotype.** Mutagenesis strategy to insert the L (I634A) missense mutation into the GR gene. GR^wt/D^ zygotes were used in CRISPR/Cas9 mutagenesis and were transferred to pseudopregnant females to obtain offspring containing the L variant either alone (GR^wt/L^) or in combination with the GR^D^ mutation (GR^wt/D+L^ mice). These heterozygous mice were further crossed multiple times to obtain homozygotes. The table illustrates that GR^L/L^ or GR^D+L/D+L^ homozygotes were never obtained, which indicates that the homozygous condition is lethal *in utero* or perinatal or shortly after birth. In the figure, we also display a cartoon of two GR monomers, binding on DNA, and homodimerizing by means of the DBD interactions and LBD interactions (in *red*). DBD, DNA-binding domain; GR, glucocorticoid receptor; LBD, ligand-binding domain.

As outlined in Figure 2, both GR^L^ and GR^D+L^ mouse lines were generated starting from GR^wt/D^ zygotes, which were generated by crossing GR^wt/wt^ females and GR^D/D^ males both on a pure FVB/NJ background. A single-guide RNA (sgRNA), as well as a mutating template containing the desired point mutation (I > A) as shown in Fig. S1, and Cas9 mRNA were microinjected in the male pronucleus of the zygotes. In addition to the GR^L^ point mutation, a silent mutation was also introduced in the PAM sequence creating a restriction site used for genotyping (Fig. S1). After injection, the embryos were transferred into pseudo-pregnant females and pups were born. One pup contained the desired GR^D+L^ mutation and others the GR^L^, which was confirmed by sequencing the genomic DNA or the GR^D^ alone. The heterozygous mutant mice were first backcrossed on an FVB/NJ background for five generations to remove possible off-target effects as much as possible. Next, a heterozygous intercross was started to yield homozygous mutants. Strikingly and in contrast to GR^D^ intercrosses, GR^L/wt^ or GR^D+L/wt^ intercrosses yielded no viable homozygous mice (Fig. 2). Further investigation revealed that the homozygous GR^L/L^ and GR^D+L/D+L^ mice die at or just after birth.

### Homozygous GR^L/L^, GR^D+L/D+L^, and GR^−/−^ display similar lethal phenotypes

**Figure 3:**
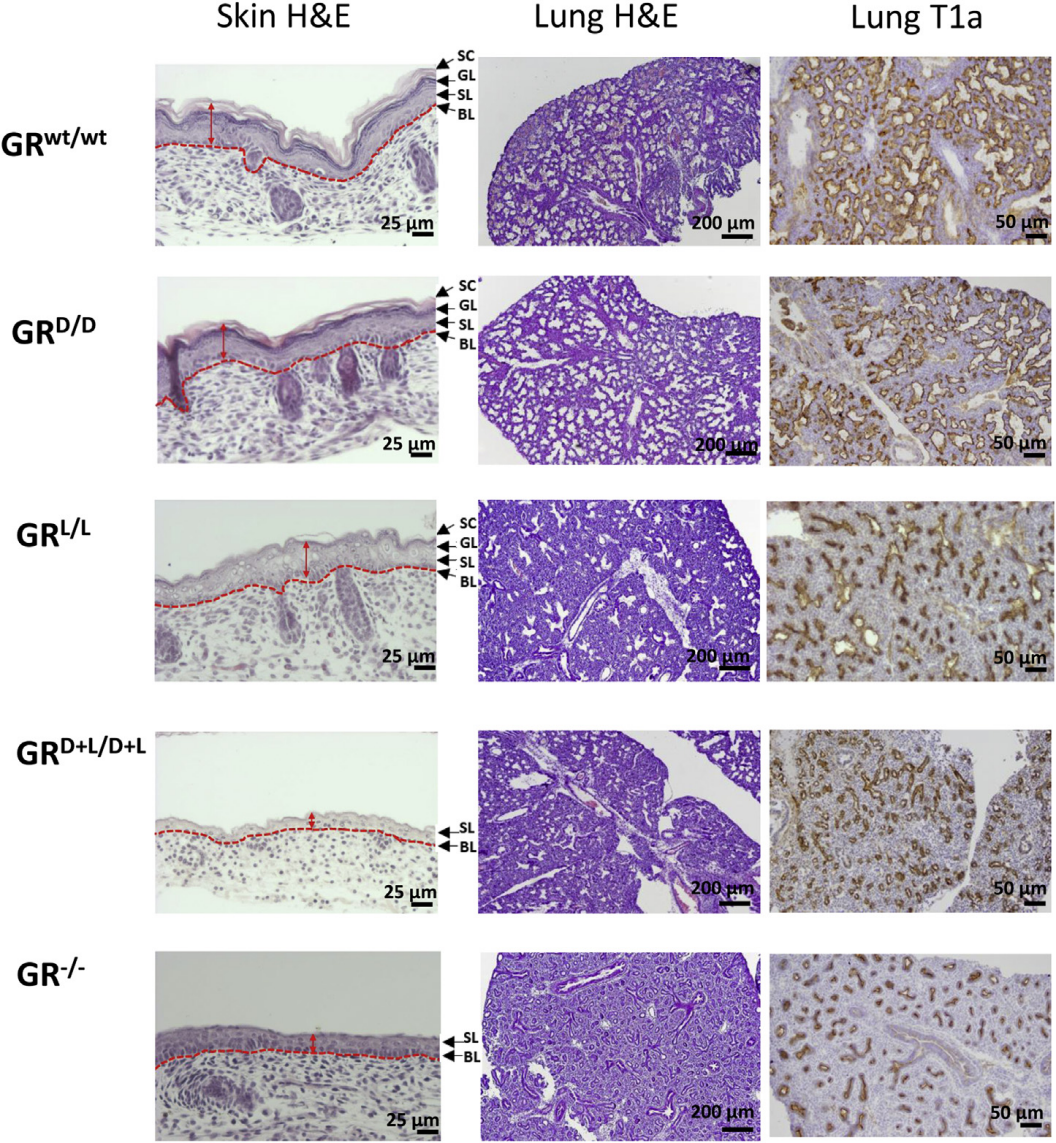
**Pathology of GR mutant mice.** Comparison of GR^wt/wt^, GR^−/−^, GR^D/D^, GR^L/L^, and GR^D+L/D+L^ at the level of the skin (*left*) and lungs (*middle* and *right panels*) on day E18,5. The GR^wt/wt^ and GR^D/D^ show a normal skin: all the skin layers are present: SC (=Stratum corneum) + GL (=Granular layer) + SL (=Spinous layer) + BL (=Basal layer). Likewise, the lungs show well developed alveoli (lung H&E), and there is high and widespread expression of the lung maturation marker T1a in the epithelial cells. The GR^L/L^, GR^D+L/D+L^, and GR^−/−^ show an abnormal skin and lung phenotype. The GR^L/L^ skin is slightly thinner and still has all layers recognizable (SC though is almost absent) but shows abnormalities in cell organization and morphology. The GR^D+L/D+L^ and GR^−/−^ have extremely thin skin with only the BL and SL recognizable. The lung H&E staining shows much denser tissue in GR^L/L^, GR^D+L/D+L^, and GR^−/−^, and the T1a marker is much less present than in GR^wt/wt^ indicating a severe lung maturation defect. GR, glucocorticoid receptor; H&E, hematoxylin and eosin.

GR^−/−^ mice die immediately after birth due to underdevelopment of the lungs (2). Lung development is normal until day of 15.5 after fertilization (E15.5), but then displays progressive hyperplasia with almost no luminal airway dilation (2, 39). In order to compare the phenotype of GR^L/L^ and GR^D+L/D+L^ with GR^−/−^, we performed histological analysis of E18.5-days-old embryos prepared after timed mating of the different genotypes and compared them with wild-type embryos. Hematoxylin and eosin (H&E) staining and immunohistochemistry (IHC) for the lung maturation marker T1a show highly similar lung development for GR^wt/wt^ and GR^D/D^ mice, with an open lung structure and high presence of the T1a maturation marker. The other mice display a compact mesenchyme, which matches the previously observed absence of luminal airway dilation in GR^−/−^, as well as a reduced presence of the T1a marker, thus lower number of differentiated lung epithelial cells and more immature, dividing cells (Fig. 3). GR^−/−^ mice have also been reported to suffer from an incompletely differentiated skin, leading to problematic fluid retention (5). The incomplete epidermal stratification observed in E18.5-days-old GR^−/−^ embryos, which was not observed in GR^wt/wt^ and GR^D/D^ embryos, was also observed in GR^L/L^ and GR^D+L/D+L^ pups, which clearly show an abnormally thin skin and absence of one or more skin layers that should present in differentiated skin (Fig. 3).

### GR function in mouse embryonic fibroblasts (MEFs)

**Figure 4:**
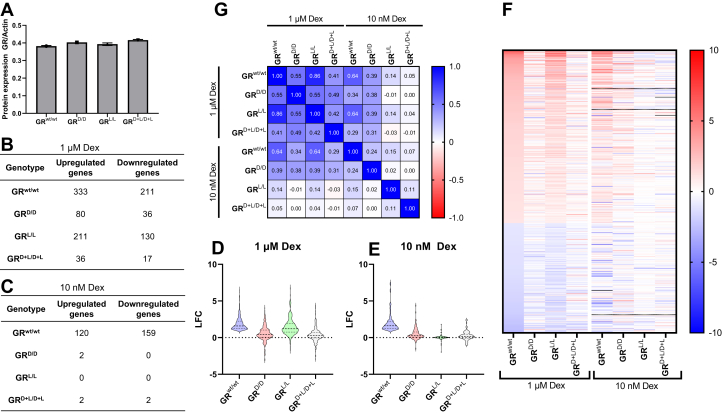
**Functional studies on the different GR mutants in MEF cells**. *A*, quantification of GR levels (FIJI using ACTIN as loading control (n = 2)) showed no significant differences in GR levels between the mutants (blot: Fig. S8). *B*, number of differentially expressed genes per genotype, DEX *versus* solvent found in the RNAseq with 1 μM Dex for 5 h (|LFC| ≥ 1; adjusted *p*-value ≤ 0.05). *C*, number of differentially expressed genes per genotype, DEX *versus*. solvent found in the RNAseq with 10 nM Dex for 5 h (|LFC| ≥ 1; adjusted *p*-value ≤ 0.05). *D*, violin plot of the magnitude of LFC values for genes upregulated by 1 μM Dex in WT in the other genotypes. The GR^wt/wt^ and GR^L/L^ show similar magnitudes of regulation. *E*, violin plot of the magnitude of LFC values for genes upregulated by 10 nM Dex in WT and in the other genotypes. The GR^wt/wt^ regulated genes show no regulation in the other genotypes. *F*, heatmap of genes regulated in all conditions, in both 1 μM Dex and 10 mM Dex experiments. Values shown are log2 fold changes, *black* indicates values outside the range on the scale (> 10 or < −10). *G*, correlation matrix between all LFC changes in Dex *versus* control, within and between the 1 μM and 10 nM Dex experiments. The nonparametric spearman correlation values were calculated and are shown in the matrix. GR, glucocorticoid receptor; LFC, log2 fold change; MEF, murine embryonic fibroblast.

E13.5-days-old embryos of heterozygous intercrossed mice were isolated and primary murine embryonic fibroblast (MEF) cells isolated, cultured, and genotyped. Homozygous GR^wt/wt^, GR^D/D^, GR^L/L^, and GR^D+L/D+L^ cells were identified, expanded, and characterized. First, it was obvious that the total amount of GR protein in all four cell types was similar in unstimulated conditions (Fig. 4*A*). We then characterized the different GR mutants in GR stimulated conditions using Dex. Dex has a high affinity for the GR and does not bind the MR, in contrast to endogenous corticosterone, which makes it perfectly suited to investigate GR only mediated effects. Based on previous work by our and other groups, we used a concentration of 10^−6^M (1 μM) Dex. We investigated nuclear translocation using confocal microscopy and found that significant nuclear translocation was induced in all genotypes. The effect size was even the largest in GR^D+L/D+L^ cells, which was somewhat unexpected (Fig. S2). The data suggest that neither the D nor the L mutation negatively impacts the initial response to high doses of ligand leading to nuclear translocation, as also suggested by Presman *et al.* (36).

Based upon these results, a genome-wide transcriptome profiling was done by RNA-seq on three MEF cultures, each one derived from a different mouse, per genotype for three biological replicates that were stimulated with either vehicle or 1 μM Dex for 5 h. Data were analyzed as described in the methods, and differentially expressed genes (with absolute log2 fold change (|LFC|) ≥ 1 and false discovery rate (FDR) ≤ 0.05) were used for further analysis. A complete overview of the number of detected genes after 1 μM DEX can be found in Figure 4*B* for all conditions. The effect of DEX in the different GR variants on gene expression gives a clear order of GR^wt/wt^, followed by GR^L/L^, which is capable of regulating many of the same genes, but not strongly. This can be seen in Figure 4*G* (1 μM Dex) where the correlation between the LFC changes in GR^wt/wt^ and GR^L/L^ is high (0.86). The GR^D/D^ performs relatively poorly, both in effect magnitude and in number of differentially regulated genes (Fig. 4, *B* and *D*). Finally, the double mutant, GR^D+L/D+L^, shows an almost nonexistent effect of Dex. We also included MEFs, which are KO for the GR (GR^−/−^) as negative controls, here no effect at all of Dex was obtained, and this condition was not included in any figures and excluded from further analysis. If the number of regulated genes in the wild type is taken as 100%, the GR^D/D^, GR^L/L^, and GR^D+L/D+L^ regulate 21%, 63%, and 10% of the genes that should be under GR control, respectively. When taking into account only the expression of *normal* (*i.e.*, by WT GR) GR regulated genes, there is also a clear difference in effect magnitude, with large fold changes observed in GR^wt/wt^ and GR^L/L^ and much lower or even opposite effects in the other genotypes (Fig. 4*D*). For technical reasons, the experiment was performed in two parts, one with GR^wt/wt^, GR^D/D^, GR^D+L/D+L^, and GR^−/−^ and a second one where we profiled the GR^L/L^ alongside GR^wt/wt^. We minimized technical variation between runs by using identical processing, including sequencing on the same machine. Still present batch effects from the setup were corrected for by making a multivariate model for differential gene expression calling where the batch effects were included as an experiment term in the formula (deseq2 formula: ∼ experiment + condition).

The primary goal was to estimate the GR dimer mediated regulatory potential of the different variants and a clear order of activity could be established. However, when further investigating other aspects using a Dex titration approach, some additional information came to light, as the behavior of the GR^L/L^ especially changed radically at low level of Dex. In order to fully understand this, a new RNAseq transcriptome profiling was performed using a lower concentration, 10 nM Dex in all genotypes. As expected, a lower number of genes were found to be differentially expressed in the GR^wt/wt^ after 10 nM Dex than after 1 μM Dex. All of the mutant GRs showed a dramatic drop in regulatory activity with the GR^L/L^ no longer able to regulate gene expression at all (Fig. 4*C*). The median LFC observed by GR activation for the gene regulated in WT shows a median LFC of around 2 in WT, in the other genotypes a median LFC of 0 is observed (Fig. 4*E*). Furthermore, while with 1 μM DEX the correlation in gene expression between GR^wt/wt^ and GR^L/L^ was high, no correlation can be found after 10 nM Dex. Here only the GR^wt/wt^ and GR^D/D^ show some level of correlation (Fig. 4*G*). Figure 4*F* shows an overview of the expression of all genes regulated by 1 μM Dex in WT GR, and what their LFC is in all the other DEX *versus*. control comparisons. This clearly shows that with high ligand concentrations, the GR^wt/wt^ and GR^L/L^ are most alike as are the GR^D/D^ and GR^D+L/D+L^. However, under low ligand concentrations, GR^wt/wt^ and GR^D/D^ are the most alike. These data show that dimer formation, especially at lower ligand concentrations, is not sufficient to explain the behavior of GR^L/L^ mutant.

### GR dimerization capacity in MEFs

**Figure 5:**
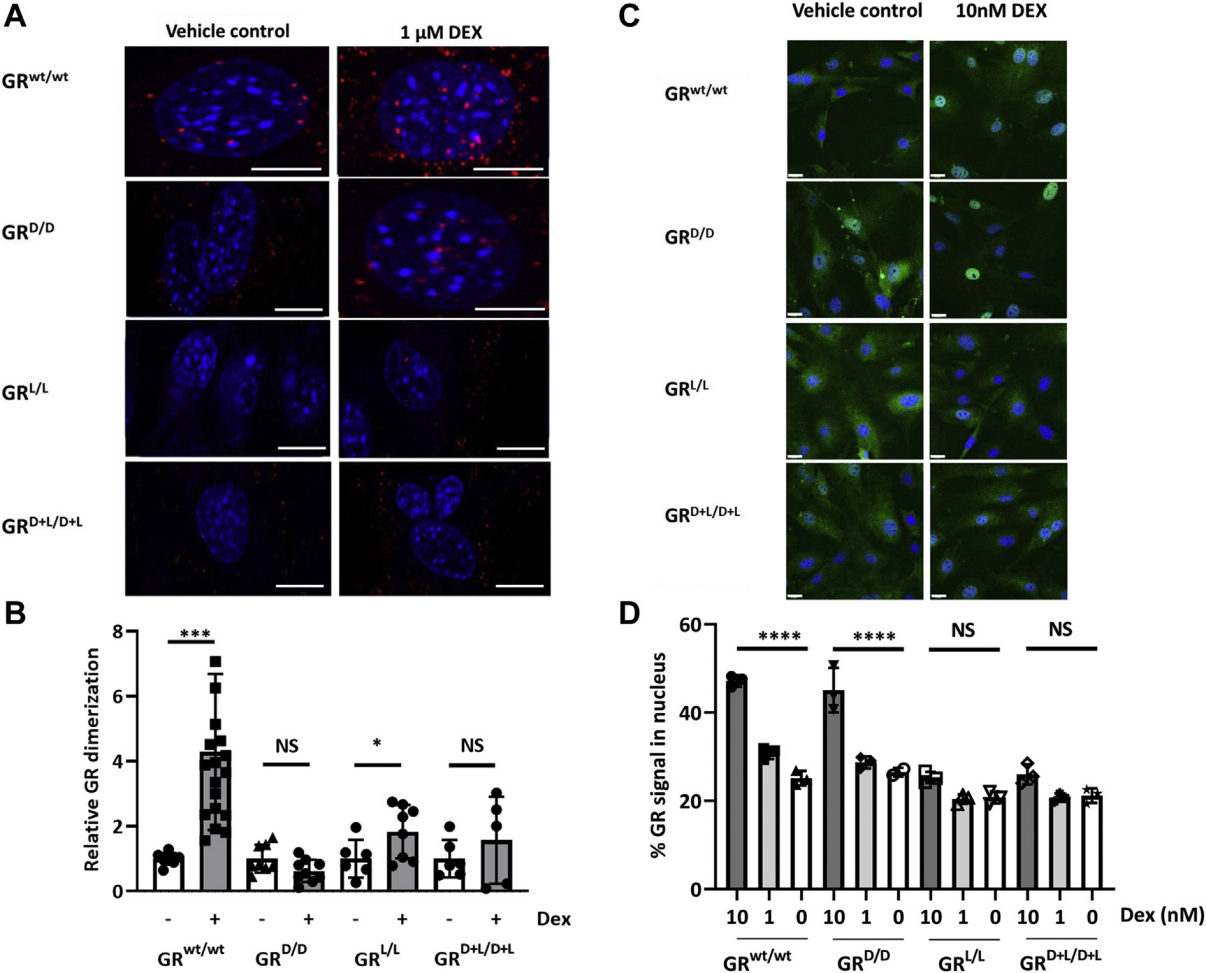
**GR homodimerization in mutant MEF cells by proximity ligation assay (PLA) and nuclear translocation of mutant GRs in MEF cells and ligand binding.***A*, representative images from the PLA assay with and without ligand (1 μM Dex). Only the GR^wt/wt^ shows a high signal (*red*) after stimulation. Scale bars are 10 μm. *B*, quantification of PLA signals in the four MEF cell types after vehicle control (*white bars*) and after Dex stimulation (*gray bars*). Group sizes were GR^wt/wt^ (N vehicle: 9; N DEX: 19), GR^D/D^ (N vehicle: 8; N DEX: 9), GR^L/L^ (N vehicle: 6; N DEX: 6), and GR^D+L/D+L^ (N vehicle: 6; N DEX: 8). Statistical significance was calculated by comparing vehicle controls with Dex effects and Student *t* test. *C* and *D*, we investigated nuclear translocation of the GR at a concentration of 10 nM and 1 nM Dex in all genotypes using IHC and confocal fluorescence microscopy. *C*, representative pictures of GR nuclear translocation after solvent and 10 nM Dex. Images shown were made by collapsing the Z-stacks using summed intensities and joining the color channels to one image. Scale bars are 22 μm. *D*, images were taken from primary MEF cells derived from three mice per genotype to obtain biological replicates and stimulated with 0, 1 nM and 10 nM Dex. A total of four Z-stacks per sample were imaged to obtain sufficient imaged cells for quantitative analysis. The plot shows the proportion of nuclear GR signal compared with total GR signal in the images. N = 3 in each condition. Signal intensities were compared with an ANOVA test using GraphPad Prism and post-hoc tests. GR, glucocorticoid receptor; MEF, murine embryonic fibroblast.

Using a proximity ligation assay (PLA), the homodimerization capacity of GR in the primary MEF cells was investigated. Cells were treated with 1 μM of Dex or with control medium (0.01% ethanol), 45 min later they were subjected to fixation, antibody binding, and PLA reaction, leading to red fluorescent signals reporting dimerization. Pictures taken by confocal microscopy were used to measure and quantify the signals. Representative pictures are displayed in Figure 5*A*, and the degrees of dimerization are shown in Figure 5*B*. Clearly, the GR^wt/wt^ MEF cells undergo strong GR dimerization after Dex treatment. After quantification, a significant increase of 4.3 × was detected in the GR^wt/wt^ cells. In this assay, both the GR^D/D^ cells and GR^D+L/D+L^ cells displayed no significant Dex-induced dimerization, while the GR^L/L^ cells had a modest but significant formation of dimers of 1.8 ×. Since this assay is performed under conditions of maximal nuclear translocation, the absent dimerization of GR^D/D^ is noteworthy, confirms the original paper reporting that GR^D/D^ does not dimerize (32) but is in contrast to later papers (36). The strongly reduced dimerization of GR^L/L^ is in line with the key role of this isoleucine in LBD dimerization described by Bledsoe *et al.* (8).

#### Nuclear translocation of the GR at physiological concentrations of ligand in MEFs

Our data suggest that at high doses of 1 μM Dex, the GR^L/L^ point mutation in the GR LBD does not prevent nuclear translocation but suffers from diminished dimerization and causes about half maximal induction of GRE genes compared with GR^wt/wt^. So, the embryonic lethal GR^L/L^ mutation appears less hampered at the level of dimerization potential and gene induction than the viable GR^D/D^ mutation. To find out why the GR^L/L^ point mutation is not viable, we studied GR nuclear translocation at lower ligand concentration. Based on Gong *et al.* (40) we estimated the endogenous GC concentration in mice to be 0.5 μM. Because Dex is up to 80 times more potent than endogenous GCs we selected 10 nM of Dex as the optimal concentration to stimulate homozygous GR mutant MEF cells. Figure 5*C* shows a representative example for each genotype 15 min after 10 nM Dex or solvent (0.0001% ethanol) control. Significant nuclear translocation was observed in GR^wt/wt^ and GR^D/D^ cells after Dex stimulation, but less in GR^L/L^ and GR^D+L/D+L^ cells (individual channels for these images can be seen in Figs. S3 and S4). After quantification (Fig. 5*D*), we observed a significant increase in nuclear GR signal in GR^wt/wt^ and GR^D/D^ cells compared with GR^L/L^ and GR^D+L/D+L^ cells. This significant difference is also found with the lower 1 nM Dex concentration, but the signal is less prominent. A significant difference between GR^wt/wt^ and GR^D/D^ or between GR^L/L^ and GR^D+L/D+L^ could never be found. In the absence of ligand, no differences between any of the genotypes were detected. These data suggest that the GR^L/L^ point mutation leads to impaired nuclear translocation, and since this occurs only at lower ligand concentrations, a possible problem of binding of the GR^L/L^ and GR^D+L/D+L^ versions to ligands is conceivable, and a possible explanation for the lethal phenotype that we observed in such homozygous mice.

#### Ligand affinity

**Figure 6:**
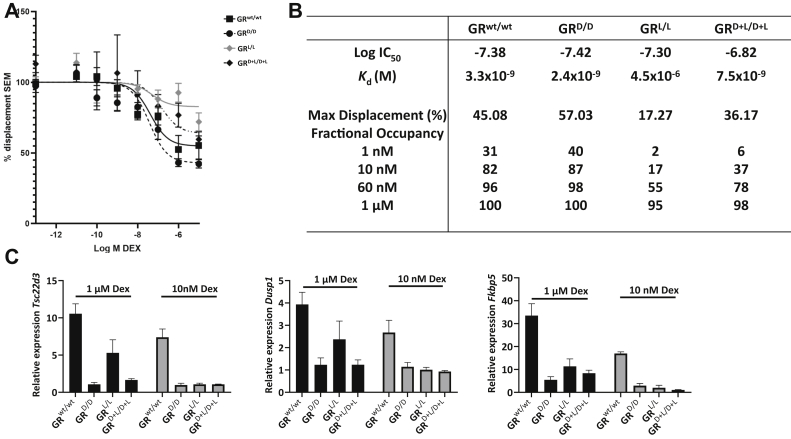
**Binding affinity of mutant GR versions to Dex and response of mutant GR to low-doses Dex.***A*, titration curves of the competitive binding assay. The GR^L/L^ does show very minimal displacement by unlabeled Dex. *B*, table with the IC_50_ values derived from the one site binding model and calculated *K*_d_ using the Cheng–Prusoff equation. The *K*_d_ and max displacement show comparable affinity for ligand in the GR^wt/wt^ and GR^D/D^ variants and 2 to 3× lower ligand affinity in the GR^D+L/D+L^ variant and about 1000× lower in GR^L/L^ cells. The calculated fractional occupancy is added to the table. *C*, induction of GRE genes *Fkbp5*, *Tsc22d3*, and *Dusp1* by 1 μM and 10 nM Dex (during 5 h incubation) in MEF cells measured by qPCR. Expression is relative compared with solvent control (0.01% and 0.0001% ethanol). GR, glucocorticoid receptor; GRE, GC response element; MEF, murine embryonic fibroblast.

Using tritiated (^3^H) Dex, GR ligand affinity was assessed using a competitive binding assay, measuring displacement of 60 nM (^3^H) Dex by increasing concentrations of unlabeled Dex, from 10^−13^ M to 10^−5^ M. The resulting titration curves and derived values for the IC_50_, max displacement and *K*_D_, which was calculated from the IC_50_ through the Cheng–Prusoff equation (41), can be found in Figure 6, *A* and *B*. We were able to successfully obtain a titration curve of ^3^H Dex replacement for all four genotypes. However, in the GR^L/L^ we observed a very weak ligand binding, much weaker than in the other MEF genotypes. The GR^wt/wt^ and GR^D/D^ show a *K*_D_ in the same range (around 2–3 nM), with the GR^D/D^ showing a slightly better affinity for Dex, and the lowest max displacement value. This indicates that the maximal effect of Dex would be the highest in the GR^D/D^, which agrees with the nuclear translocation experiment at 1 μM Dex, where a better nuclear translocation signal was found in GR^D/D^ compared with GR^wt/wt^ cells (Fig. S2). The GR^D+L/D+L^ shows reduced displacement compared with the GR^wt/wt^ and GR^D/D^, and a *K*_d_ value of 7 nM. This is a minimal twofold higher *K*_d_ than the GR^wt/wt^ and GR^D/D^ but clearly not good enough to sustain survival of mice. In addition, the GR^D+L/D+L^ has a poor displacement value, where only 36% of the labeled DEX could be displaced at saturation compared with 45% and 57% for the GR^wt/wt^ and GR^D/D^, respectively. The poorest affinity was observed with the GR^L/L^ mutant. Calculating the *K*_d_ was difficult due to the extremely low maximal displacement, with only 17% of the ^3^H Dex being replaced at saturation. An accompanying *K*_d_ of 4.5 μM could be derived for this effect.

In addition, based on calculated *K*_d_ values, we were able to predict the fractional occupancy (FO) at various ligand concentrations (Fig. 6*B*) using the formula $FO=\frac{\left[L\right]}{\left[L\right]\times K_{d}}$ and applied this to the ligand concentration used in our nuclear translocation experiments, 10 nM and 1 μM of Dex. At the low concentration of 10 nM of Dex, the GR^wt/wt^ and GR^D/D^ receptors show a comparable fractional occupancy of 82% and 87%, respectively. However, the fractional occupancy for the other mutants, which do not undergo strong evidence of nuclear translocation at this concentration, is much lower with the GR^D+L/D+L^ showing 37% and GR^L/L^ showing 17% fractional occupancy. At the high, 1 μM, concentration we calculate that the GR^wt/wt^ and GR^D/D^ receptors are fully saturated with a fractional occupancy of 100% and that the GR^D+L/D+L^ is almost completely saturated (98%) as well as the GR^L/L^ at 95%, but when comparing with the effect of lower Dex concentrations on GR^wt/wt^ or GR^D/D^ and related fractional occupancy, this is still more than sufficient to allow for normal nuclear localization. The hypothesis of reduced ligand binding by the GR^L/L^ mutation was strengthened by qPCR analysis of induction of several GRE genes (*Fkbp5*, *Tsc22d3*, and *Dusp1*) by 1 μM and 10 nM Dex in MEF cells (Fig. 6*C*). In accordance to the RNAseq data at 1 μM Dex and several qPCR experiments at multiple Dex concentrations, we found that the better performance of GR^L/L^ compared with GR^D/D^, which is typical at the high Dex dose, is completely lost at the lower Dex dose.

## Discussion

GR variants with impaired dimerization have proven to be invaluable tools to study the various receptor mechanisms of action in physiology and disease, to develop synthetic GCs and to understand glucocorticoid resistance (42, 43, 44). A GR with a disruptive mutation in the primary dimerization interface in the DBD, the GR^dim^ version expressing an A465T mutation, has been generated in 1998 (32). As this GR^D^ receptor displayed residual dimerization propensity, an additional mutation was introduced, namely I634A located in the LBD (8, 36). Based on *in vitro* cellular transfection studies, both point mutations present in one single GR protein (the so-called GR^mon^ mutation) appeared to reduce GR dimerization to virtually undetectable levels (36). However, until now this GR^mon^ variant has not been investigated at all in an *in vivo* model and a mutant GR having only I634A substitution has not been generated and investigated. To understand the *in vivo* consequences of the addition of the LBD mutation to the DBD mutation, we have generated mice that exhibit the I634A mutation, either alone (GR^L^ mutation) or in combination with the A465T mutation, the GR^D+L^ mouse. Our motivation to replace the isoleucine on position 634 by an alanine was based on the previous *in vitro* studies mentioned, but also because in some mammals the only substitution found at this position is a valine (Fig. S5). These mice were generated with the idea to study pathophysiological aspects and responses to (endogenous) ligands in homozygous mutant mice. A summary of the findings of this paper is provided in Table S2.

After obtaining hundreds of offspring, no single homozygous GR^L/L^ or GR^D+L/D+L^ mouse was born alive. The phenotype observed in embryos of both homozygous mouse lines was found to be identical to what was previously described in the full GR^−/−^ genotype, *i.e.*, a clearly underdevelopment of skin and lungs, leading to problematic fluid homeostasis and breathing (2). So, *in vivo*, these variants appear to result in full loss of function of the GR, despite that these GR versions had previously been described to have functions *in vitro*, including ligand binding, translocation, and transcriptional repression activities (20, 32, 36). We were puzzled whether the lethality of GR^D+L/D+L^ could be due to the complete absence of dimer formation of this variant, because the main DBD dimerization interface in the GR^L/L^ mutants was still supposed to be intact while GR^L/L^ mice were not viable either.

The double point mutant GR^D+L^ and single GR^L^ mutant had been shown previously to display full nuclear translocation using a high dose of Dex (0.1–1 μM) in an *in vitro* cell transfection system (36). Using primary cultures of untransformed MEF cells derived from embryos, we confirmed this finding of full nuclear translocation using 1 μM Dex. The subsequent profiling showed that the GR^D+L/D+L^ cells performed very poorly on both transcriptional activation GR responsive genes and of dimerization. Although a further reduction in dimerization in the double mutant cells compared with GR^D/D^ could not be demonstrated, the double mutation led to another 50% (from 20% gene expression increase compared with controls to 10% gene expression increase) reduction of gene induction capacity of GRE genes compared with GR^D/D^ cells, which already showed only fraction of activity compared with GR^wt/wt^ cells. These results are in agreement with the recently published study by Johnson *et al.* (45), where an in-depth *in vitro* comparison of the GR^wt/wt^, GR^D/D^, and GR^D+L/D+L^ genotypes was performed. However, we found that GR^L/L^ cells displayed a significant residual dimerization as well as only a 50% reduction on dimer mediated transcriptional activation as compared with GR^wt/wt^. This means that, despite the lethal phenotype, the GR^L/L^ performs second best in GR mediated gene regulation, next to the normal WT receptor. In our studies, and given the near physiological relevance of our system, we did find however a complete lack of dimer formation in GR^D/D^ MEF cells, which again supports that the DBD dimerization interphase does contribute very significantly to GR homodimerization (32), in contrast to other studies (36).

The crystal structure of the GR LBD revealed that I634 is one of the six essential amino acids involved in GR dimerization mediated by the LBD (8) (Fig. 1). Our studies confirm the importance of this residue in dimerization in saturating conditions of ligand. The LBD crystal structure has also identified the amino acids that are involved in interaction with the ligand (Fig. 1*B*). Although I634 is not one of these amino acids, it has been suggested that the GR^I634A^ variant may impact the ligand-binding capabilities to some extent (8), as it is located close to the key ligand-binding residues (Fig. 1*B*). Based on the nonviability of the GR^L/L^ and GR^D+L/D+L^ mice, we performed nuclear translocation experiments at physiological concentrations of GCs based on values from peak diurnal GC levels in mice in the absence of stress. We did consider using endogenous GCs in our essays but ultimately decided that the disadvantages were to great: (i) endogenous GCs are not specific to the GR and also bind and activate the MR adding an important confounding factor to any measurements. (ii) Saturation that was used for some assays is easier with a more potent ligand, and such ligand can be easily diluted when needed. The Dex compound also exhibits some useful pharmacological properties (*e.g.*, low breakdown long-lasting). We found that the GR^L/L^ and GR^D+L/D+L^ cells displayed almost no nuclear translocation of the receptor at physiological GC levels, whereas the GR^wt/wt^ and GR^D/D^ show no significant differences to one another and translocated normally to the nucleus. Moreover, the significant drop in Dex response toward gene induction of the GR^L/L^ cells at lower Dex dose is very remarkable and compatible with a poor ligand binding.

**Figure 7:**
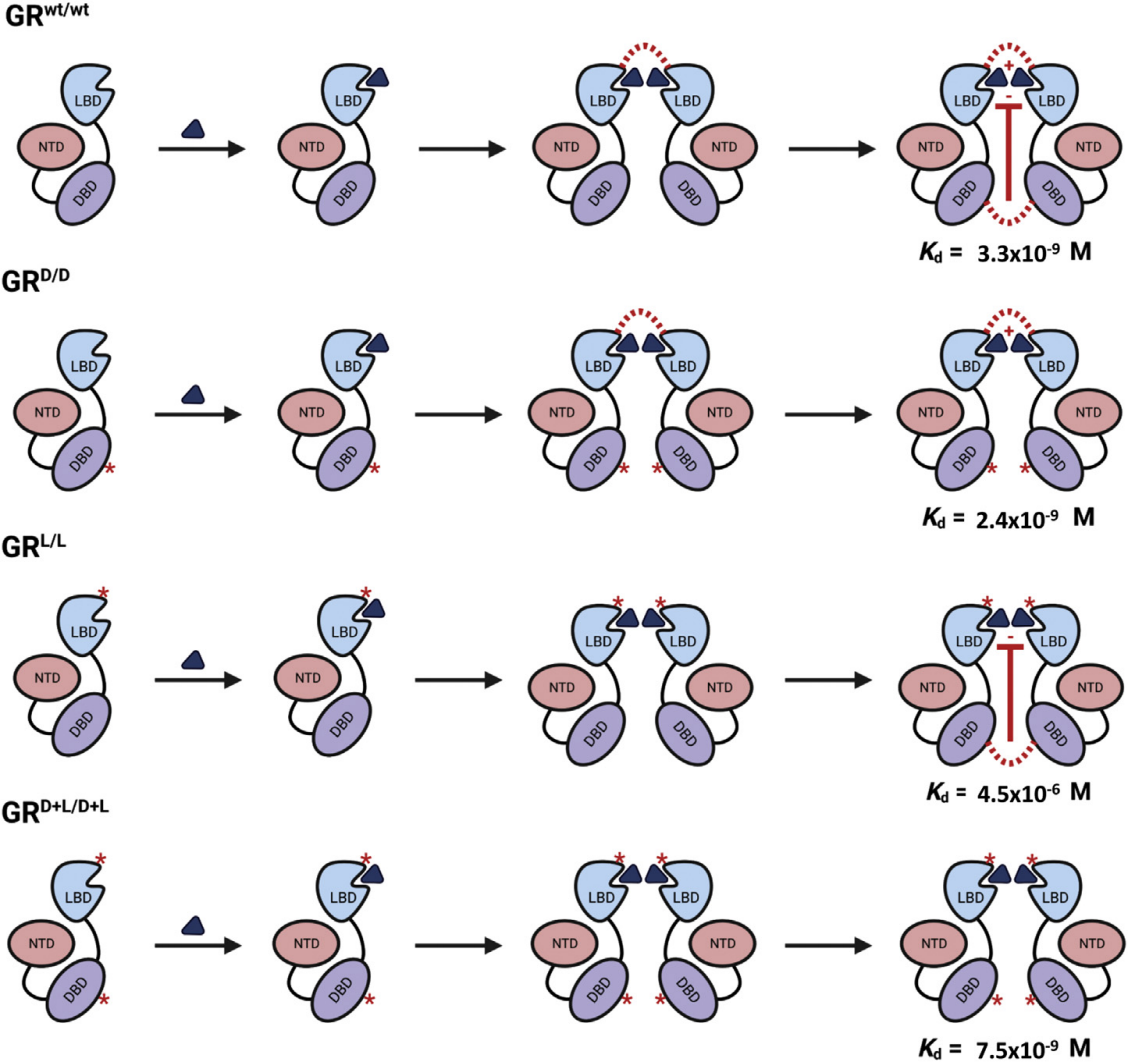
**Hypothesis explaining binding affinities of point mutant GR molecules.** In GR^wt/wt^ cells, ligand (the *blue triangle*, Dex) binds and GR monomers dimerize by interactions between monomer LBDs and DBDs (*red dotted lines*). Since the *K*_d_ of ligand binding in GR^D/D^ cells is lower than in GR^wt/wt^ cells, a negative cooperative effect from the DBD toward ligand binding (drawn as the *red line* and – symbol) is conceivable in GR^wt/wt^ cells and absent in GR^D/D^ cells. Since the *K*_d_ in GR^L/L^ cells is the highest of all conditions, the negative cooperative effect is obvious, but also the lack of a positive cooperative effect (drawn as a + symbol), which is thus provided by the DBD dimerization. Ligand binding in GR^D+L/D+L^ cells leads to undetectable dimerization and to a *K*_d_, which is a magnitude lower than in GR^wt/wt^ cells. DBD, DNA-binding domain; GR, glucocorticoid receptor; LBD, ligand-binding domain.

Next to the reduction in dimerization potential, we provide proof of lower ligand affinity in the GR^L/L^ and GR^D+L/D+L^ mutants. We found a slightly lower *K*_d_ in GR^wt/wt^ than GR^D/D^ and at least 1000 × less specific binding in GR^L/L^. In GR^D+L/D+L^ mutants, a *K*_d_ 2.5 × lower compared with GR^wt/wt^ was detected. These differences in ligand affinity remain unexplained, but they might suggest that the DBD dimerization provides a ligand-affinity lowering signal, while the LBD dimerization causes an opposite signal (Fig. 7). The exact nature of these signals is not known. It is accepted that ligand binding transduces a signal to the DBD for increased dimerization (33). Conceivably, the reverse could also be possible.

These results are largely compatible with what is shown in the recent *in vitro* study of the GR mutants, where it is shown that the transcriptional response is much reduced in GR^D/D^ and almost absent in GR^D+L/D+L^ (45). Indeed, as the GR^wt/wt^ and GR^D/D^ behave normally in ligand binding and translocate to the nucleus, the differences in expression can only be explained by differences in DNA binding between both receptors. Johnson *et al.* (45) propose that the differences in DNA binding are caused by higher-order receptor interaction and through the chromatin state of the cells, where some closed chromatin regions can be bound by GR^wt/wt^ but is restricted to open chromatin GR^D/D^. However, we propose that for the GR^D+L/D+L^, as well as for the GR^L/L^ alone, reduced ligand–receptor interaction contributes significantly the lethality and can also in large part explain the very low DNA-binding observed by Johnson *et al.* for the GR^D+L/D+L^. A low level of GR in the nucleus will also prevent DNA binding and give very low signal intensities (45).

The exact reasons for the reduction in ligand biding are not yet known, but we could suggest that (i) ligand binding is affected directly by changes in the structure of the LBD, possible making it more difficult to bind or easier to dissociate. We note that, although not directly interacting with ligand, I634 resides on the opposite face of a beta-hairpin that does interact with ligand (8). It is conceivable that structural changes caused by I634A at the LBD dimer interface may propagate into the ligand binding to compromise ligand-binding affinity and make the LBD–ligand complex less stable. Or (ii) the I634A mutation may change the GR interaction with other proteins (cofactors) that bind to the LBD. (iii) In the LBD dimer formation, the I634 is part of the core hydrophobic domain. Indeed, the major function of this I may reside in its hydrophobic interaction with the P632 of the other monomer as shown by Bledsoe. This P is one of the six crucial amino acids in the dimerization of the LBDs. Removal of the hydrophobic side chain appears to be sufficient to cause lethality. A multispecies comparison shows that an I→V substitution, and only this one, occurs in nature and results in functional receptors (Fig. S5). The A is (much) less hydrophobic than the naturally found valine and isoleucine. It is possible that these hydrophobic interactions are needed for more than just dimer formation but are important for the LBD structure and/or general protein–protein interactions (*e.g.*, with chaperones). This is also in line with results from known pathological variants in the LBD, all of which affect nuclear translocation, ligand affinity, and/or GR–cofactor interactions (46) (Fig. 1).We applied two tools, PROVEAN and SIFT, which evaluate the impact of an AA substitution of protein function, and both tools confirm that a V may be accepted, but not an A or even a L, on this I position (Fig. S6). The I at position 634 in mouse is found as an I at position 628 in humans (but as a V in other species). The V results in a normally functioning receptor and is indeed considered a conservative mutation from a structural standpoint: based on the human LBD crystal structure (8), the hydrophobic core of the LBD dimer interface is comprises 2-stranded beta-hairpins and an I → V is the most conservative substitution for amino acids found in beta-sheets. The closely related amino acid L is never found at the position in any species. Upon examination of the structure, an L on 628 (human) would create steric hinderance with another residue (Cys on 622) from the same GR chain in the domain, preventing the hydrophobic pocket from being formed, which would probably result in the same lethal phenotype as our I→A mutation. The A residue that was substituted in the GR^L^ variant is too short to interact with the normal Pro binding partner (P625) from the other GR chain, so the hydrophobic interface cannot be formed, resulting in loss of function (Fig. S7). Finally (iv) there may also be contributing signal from higher-order structures, the presence of which cannot be excluded by our assay. It has been shown that the GR may form tetramers, which have an important impact on occupancy and transcriptional activity. The I→A may also disrupt the forming of such structures, resulting in negative effects. However, based on the location of the amino acid in the well-known dimer structure, we believe that a direct role in the formation of higher-level structures is unlikely, but may be potentially investigated by combining with the described tetramer inducing (GRtetra) mutation. The presence of such higher-order structures and any difference in their occurrence, detectable or not, does not impact our conclusions of the LBD mutant: in low ligand concentrations, it does not bind ligands and consequently does not go to the nucleus where any GR structure, be they dimers or tetramers, may be formed.

We propose that the lower ligand affinity can be solely responsible for the lethal phenotype of both homozygous mutants containing the I634A mutation. The use of high doses of Dex compensates for lower ligand affinity in the MEF cell system. At such saturating ligand levels, the GR^L/L^ cells display the best performance of all three point-mutant cells. In the GR^L/L^ mice, we believe that the reduced ligand affinity of the receptor is the primary driver of the neonatal lethality. The impaired ability of the receptor to bind its ligand at low concentrations effectively prevents nuclear translocation and abrogates GR mediated transcriptional processes creating a phenocopy of the GR^−/−^ mice.

In the GR^D+L/D+L^, the absence of nuclear translocation is conceivably also the driving factor for lethality, but here even the presence of nuclear translocation may matter less since the transcriptional performance of this mutant is so low that it may still create developmental problems even in the case that nuclear translocation would occur. An initial set experiments aiming to rescue the birth of homozygous GR^L/L^ mice using Dex injections and Dex in drinking water of pregnant mothers have failed. However, a follow-up using different doses and treatment start times may be informative as Dex can also be toxic to development if given early.

Our work confirms the hypothesis that the GR^L^ mutation results in reduced affinity for ligand, as was proposed by Bledsoe *et al.* (8) based on structural studies in the human GR LBD. Mutagenesis in the LBD to test the impact of relevant amino acids on the LBD dimerization interphase may reveal data *in vitro*, but may fail to yield viable homozygous mice, because point mutations in the LBD have a high chance of disturbing physiological function of GR at the level of ligand binding. Whether the findings with the I634A residue change would lead to similar findings (importance for dimerization but also ligand affinity) (1) when mutating it to another amino acid (*e.g.*, I634G), or (2) when addressing other amino acids in the vicinity, can only be guessed for the time being.

Since both GR^L/wt^ and GR^D+L/wt^ mice are perfectly healthy, our data confirm that homozygous mutations in the LBD are generally very poorly tolerated as they often impact the functioning of the domain, but several heterozygous ones may be viable and thus be discovered. So, in humans, almost all GR mutations occur in heterozygous condition, and even then, they can cause GCR phenotypes. In Figure 1, we project these observed human sequence variations on the mouse *Nr3c1* gene. The few that are found in homozygous condition (V577A and G685S) allow viability but result in severe phenotypes resulting from glucocorticoid resistance (*e.g.*, glucose levels, infertility, hypertension) often presenting very early (46, 47).

In conclusion, an *in vitro* well-known mutation in the LBD of GR, I634A, is not tolerated *in vivo*, leading to perinatal lethality, conceivably because it leads to insufficient ligand binding of GR. The study does confirm, however, that the I634A mutation indeed also leads to reduced DNA binding and reduced gene expression, which is compatible with a function of this AA in ligand binding as well as dimerization of GR.

## Experimental procedures

### Generation of mutant mice

The original GR^dim^ mice, officially named as Nr3c1^tm3Gsc^, have been described in 1998 (32). It concerns a A465T mutation and was called GR^dim^, because the expected impact on GR was thought to be reduced dimerization. We are renamed this GR^dim^ to GR^D^, because we wanted to develop other dimerization defective mice. We generated mice expressing a I643AGR, called GR^L^ and mice expressing both D and L point mutations in the same GR protein, and called this mutation GR^D+L^. The mice were generated by a CRISPR/Cas9 strategy. We crossed GR^wt/wt^ females and GR^D/D^ males (both in a pure FVB/NJ background) and isolated GR^D/wt^ zygotes. These zygotes were microinjected with Cas9 mRNA (Sigma) together with a crRNA/tracrRNA duplex (IDT) with guide sequence 5′ GCTATGCTTTGC TCCTGA 3′ and single-stranded DNA oligo with sequence 5′G GCATTTGCCCTGGGTTGGAGATCATACAGACAAGCA AGTGGAAACCTGCTATGCTTTGCTCCTGATCTGATTA TTAATGAGTAAGTTACATGGCCTTAACCCTCCACAAA GAACTA 3’ (IDT) containing the I to A mutation (ATT > GCT (I643A)) and a silent mutation, mutating the PAM site (AGG > AAA) and introducing a *MluCI* restriction site for genotyping purposes. After injection, the embryos were incubated overnight in KSOM medium (Sigma-Aldrich) and transferred to foster mothers the next day through oviduct transfer. GR^dim/wt^ mice (on an FVB/NJ background) and GR^+/−^ mice (on a 129/SvJ background) were provided by Dr Jan Tuckermann (Ulm University).

### Mouse crosses and animal experimentation

GR^+/−^ mice (129, Sv) were intercrossed to obtain GR^+/+^, GR^−/+^, and GR^−/−^ mice. The GR^D^ colony was maintained by intercrossing GR^D/wt^ mice. Newborn mice from the mutagenesis program were genotyped. Mice containing a GRL allele or a GRD+L allele were found and were first backcrossed into FVB/NJ for five generations to reduce the amount of possible off-target CRISPR/Cas defects. Heterozygous mice were intercrossed to obtain homozygous offspring. To generate embryos for pathological investigation and to start MEF cultures, heterozygous couples were crossed, vaginal plugs were observed, and embryos were obtained by Caesarean section at E13.5 (to isolate MEF cultures) and E18.5 for lung and skin isolation. The day that the vaginal plug was found was considered as E0.5. All offspring was genotyped by PCR on genomic DNA isolated from toe biopsies or tails (in case of embryos). Mice were kept in individually ventilated cages under a 12 h dark/light cycle in a specific pathogen free (SPF) animal facility and received food and water ad libitum. All animal experiments were approved by the ethical committee for animal welfare of the Faculty of Sciences, Ghent University.

### Histological analysis

Lungs and dorsal skin were sampled from E18.5 embryos, fixed in 4% paraformaldehyde, and embedded in paraffin. Section of 5 μm thick were cut. For histopathology, lung and skin sections were stained with H&E using standard protocols. Pictures were taken with an Olympus B × 51 light microscope. For immunohistochemistry (T1a staining) tissue sections were dewaxed, dehydrated, incubated in TE buffer at boiling temperature. Endogenous peroxidase activity was blocked by incubating the slides in H_2_O_2_ for 10 min. Blocking buffer (3% BSA in 1 × PBS) was added to the slides for 10 min. Primary antibody against T1a (T1a: DSHB, T1a 8.1.1 ascites (https://dshb.biology.uiowa.edu/8-1-1) was diluted 1:250 in blocking buffer and incubated overnight at 4 °C. The next day, slides were incubated with secondary antibody, diluted 1:500, (anti-rabbit Immunoglobulins-HRP, Dako) for 1.5 h. Signal was visualized by incubating slides with DAB (Fluka, #32750) and counterstained with hematoxylin.

### Cell culture

MEFs were isolated from mouse embryos at day E13.5. After removal of head and organs, the embryos were minced into very small pieces with sterile surgical blades and pressed several timed through an 18-gauge needle. The cells were cultured in DMEM (Dulbecco’s modified Eagle’s medium, Gibco, Life Technologies) supplemented with 10% FCS, 1 mM sodium pyruvate, 0.1 mM nonessential amino acids, 2 mM L-glutamate, 0.2% β-mercapto-ethanol, 0.1 mM pen/strep, and 0.2% prophylactic Plasmocin at 37 °C and 5% CO_2_.

### Nuclear translocation

MEF cells were seeded in a μ-Slide (chambered coverslip) with eight wells and stimulated with 1 μM Dex, 10 nM Dex, 1 nM Dex, or solvent for 15 min. For fixation, half of the medium was replaced by 4% PFA (37 °C) and incubated for 5 min. Next, all fluid was replaced with 2% PFA for 15 min. After fixation, cells were treated with 0.1% Triton X-100 to permeabilize the nuclear membrane for 10 min. Cells were then washed with PBS and incubated with medium containing goat serum (1:500 in PBT buffer, which is PBS containing 0.1% Tween 20) for 1 h. Primary antibody against GR (GR (G-5) sc-393232, Santa-Cruz, Bio-connect; 1:1000 in PBT) was added overnight at 4 °C. Next day, the cells were washed in PBT and incubated with secondary antibody (Dylight 488 GAM IgG, Thermo Scientific 35,502, 1:1000 in PBT) for 2 h. Counterstaining was done using Hoechst (Sigma-Aldrich NV, 1:1000 in PBT) for 20 min. After washing the cells with PBT, they were mounted in 1% n-propyl gallate and visualized using a Zeiss LSM880 Fast Airyscan. Z-stacks of each genotype were taken to obtain images of minimally 30 cells per sample. Raw images were processed in the Volocity program to normalize and perform signal quantification: voxels and signal intensities were determined across two channels: one for nuclei (blue) and one for GR immunostaining (green). The voxels were merged into volumes, where the nucleus was defined as the total 3D area defined by the blue voxels. For GR channel voxels were also merged to larger volumes. Volumes smaller than 1 μm were excluded from further analysis. Nuclear GR was defined as all green voxels present in the nuclear volume (number and signal intensities), and total GR signal was the number of voxels and signal intensities in all green volumes. We performed a two-way ANOVA with post-hoc tests to detect significant differences

### Western blot

Cells were lysed in E1A buffer (50 mM HEPES pH7.6, 250 mM NaCl, 5 mM EDTA, and 0.5% NP-40) by incubating for 10 min on ice. After centrifuging, the supernatant was collected in new Eppendorf tubes, and protein concentrations were measured using Bradford. Protein samples containing 50 μg protein were separated by electrophoresis in a 10% gradient SDS polyacrylamide gel and transferred to nitrocellulose membranes (pore size 0.45 μm). After blocking the membranes with 1:2 dilution of Starting Block/PBT (Thermo Fisher Scientific), membranes were incubated overnight at 4 °C with primary antibody to detect GR (G-5) (sc-393232, Santa-Cruz, Bio-connect) or β-actin (MA5-15739, Pierce). Blots were washed with PBT and then incubated for 1 h at room temperature with secondary antibody, anti-mouse antibody (926–32,220; Li-Cor), and immunoreactive bands were detected using ECL (Amersham).

### Proximity ligation assay to detect GR dimerization

MEF cells endogenously expressing different GR proteins were treated with 1 μM Dex or vehicle control (0.01% EtOH) for 45 min. After treatment cells were fixed using 4% paraformaldehyde in preparation for the PLA using the Duolink *In situ* reagents from Sigma-Aldrich. The PLA plus and minus oligonucleotides were conjugated to a monoclonal GR antibody purchased from Santa Cruz Biotechnology (GR-F10; sc-376426). Fixed cells were blocked for 60 min with the blocking solution provided and subsequently incubated for 2 h with the conjugated antibodies. This was followed by two wash steps after which the cells were incubated with ligase to facilitate the ligation of the plus and minus oligonucleotides. The annealed oligonucleotides served as a template for the production of numerous DNA circles after incubating the cells with a polymerase solution, which also included a labeled complementary oligonucleotide probe that generates a fluorescent signal. The signal was visualized as a fluorescent dot using the LSM780 confocal microscope with ELRYA PS1 super-resolution platform (Zeiss). To visualize the nucleus, the fixed cells were stained with 10 μg/ml Hoecht33258 stain (Sigma-Aldrich). The PLA signal in the nucleus only was quantified using ImageJ software. Data were expressed as means ± SD, and statistical analysis was performed using a Student’s *t* test.

### Homologous competitive binding

Cells were plated into 24-well at a density of 5 × 10^4^ cells/well and allowed to reach 60 to 70% confluency at which point cells were steroid starved in DMEM culture medium supplemented with charcoal-stripped FCS for 24 h. Following steroid starvation, cells were washed three times with PBS and incubated in DMEM containing 60 nM ^3^H-Dex (specific activity (SA) of 71 Ci/mmol from PerkinElmer) and solvent or increasing concentrations of unlabeled Dex (10^−11^ M to 10^−5^ M) at 37 °C for 6 h.

After incubation, cells were placed on ice and washed three times with ice-cold 0.2% PBS-BSA at 4 °C, for 15 min per wash, to remove unbound ligand, and then washed with ice-cold PBS to remove albumin. Cells were then lysed with 100 μl passive lysis buffer (0.2% (v/v) Triton, 10% (v/v) glycerol, 2.8% (v/v) TRIS-phosphate-EDTA, and 1.44 mM EDTA) at room temperature and allowed to go through a freeze–thaw cycle. Thawed lysates (80 μl/well) were added to 1 ml of scintillation fluid/well (Thermo Scientific), and the counts per minute (cpm) were measured using a Tri-Carb 2810TR scintillation counter (PerkinElmer). Results were normalized to protein concentration using the BCA protein determination method (Thermo Scientific) and presented as percentage specific binding. Data were fitted to the one site binding model using Prism 9 to derive the IC_50_, which was used to determine the *K*_d_ of Dex for the GR in each MEF cell line employing the Cheng–Prusoff equation. All experiments were tested for ligand depletion and counting efficiency (CE), which was less than 10% and approximately 46%, respectively.

### RNAseq

MEF cells were seeded in 6-well plates in supplemented DMEM. Cells were treated for 5 h with 1 μM Dex or solvent. A second experiment was performed to obtain additional transcriptome data using the same setup with stimulation of either 10 nM DEX or solvent. Total RNA was isolated from solvent- or Dex-stimulated MEF cells with Aurum total RNA mini kit (732-6820; Bio-Rad) according to the manufacturer's instructions. RNA concentration was measured, and RNA quality was checked with the Agilent RNA 6000 Pico Kit (Agilent Technologies). Library preparation and sequencing were performed according to the manufacturer’s instruction with the illumina Truseq library prep kit, and sequencing was done on an illumina nextseq 550 (1 μM) or an illumine novaseq 6000 (10 nM). Data were mapped to the mouse (mm10) reference genome transcriptome with hisat2 (48). Only uniquely mapped reads were retained. Gene level read counts were obtained with feature Counts (49) (subread package). Differential gene expression was assessed with the DESeq2 package (50). Transcription factor-binding sites on collections of transcripts were identified with the HOMER (v4.6) software (51) and its accompanying collection of tools. RNA-seq reads and processed data (count matix) were deposited in GEO (GSE189356 and GSE189620)

### qPCR analysis

MEF cells were seeded at 500.000 cells per 6-well plate and stimulated with 1 μM, 10 nM, or 0 nM Dex for 5 h. Then, RNA was isolated with Aurum total RNA mini kit, and RNA concentration was measured with the Nanodrop 1000 (Thermo Fisher Scientific), and 1 μg RNA was used to prepare cDNA with Sensifast cDNA Synthesis Kit (BIO-650504, Bioline). qPCR was performed using the Roche LightCycler480 system (Applied Biosystems). The best-performing housekeeping genes were *Rpl* and *Ubc* and were determined by Genorm51. Results are given as relative expression values normalized to housekeeping genes and scaled to the geometric mean. Primers used for qPCR are depicted in Table S1.

### Statistics analysis

All data were analyzed with GraphPad Prism, except for the sequencing data as indicated before. Data are represented as mean ± SD. Statistical differences between groups were calculated by means of Student’s *t* test, one-way ANOVA, or two-way ANOVA, as indicated in the figure legends. Samples were assumed to be normally distributed with similar variance between groups. Group sizes were determined based on previous experience. No data were excluded from the analyses.

## Data availability

All sequencing datasets (RNAseq) were deposited in the Gene Expression Omnibus database (GEO) with identifiers GSE189356 for the stimulation with 10 nM Dex and GSE189620 for the stimulation with 1 μM Dex. Separate channels from the microscopy images are included in the supplemental data, the raw images, as Z-stacks obtained from the confocal microscope in czi image format are stored and are available on request. The Western blot used for the quantification in Figure 4*A* is included as Fig. S8. All other data are either directly available in the manuscript or can be provided on request.

Data requests should be communicated to the corresponding author claude.libert@ugent.be or to steven.timmermans@irc.vib-ugent.be

## Supporting information

This article contains supporting information.

## Conflict of interest

The authors declare that they have no conflict of interest with the contents of this article.
