# Supplementary material for: Point mutation I634A in the glucocorticoid receptor causes embryonic lethality by reduced ligand binding
Source: J Biol Chem. 2022 Jan 8;298(2):101574. doi: 10.1016/j.jbc.2022.101574 (PMC8808175; doi:10.1016/j.jbc.2022.101574)
Supplement: Supplemental Figures S1–S8 [file mmc1.pdf]

Supplemental Figure 1

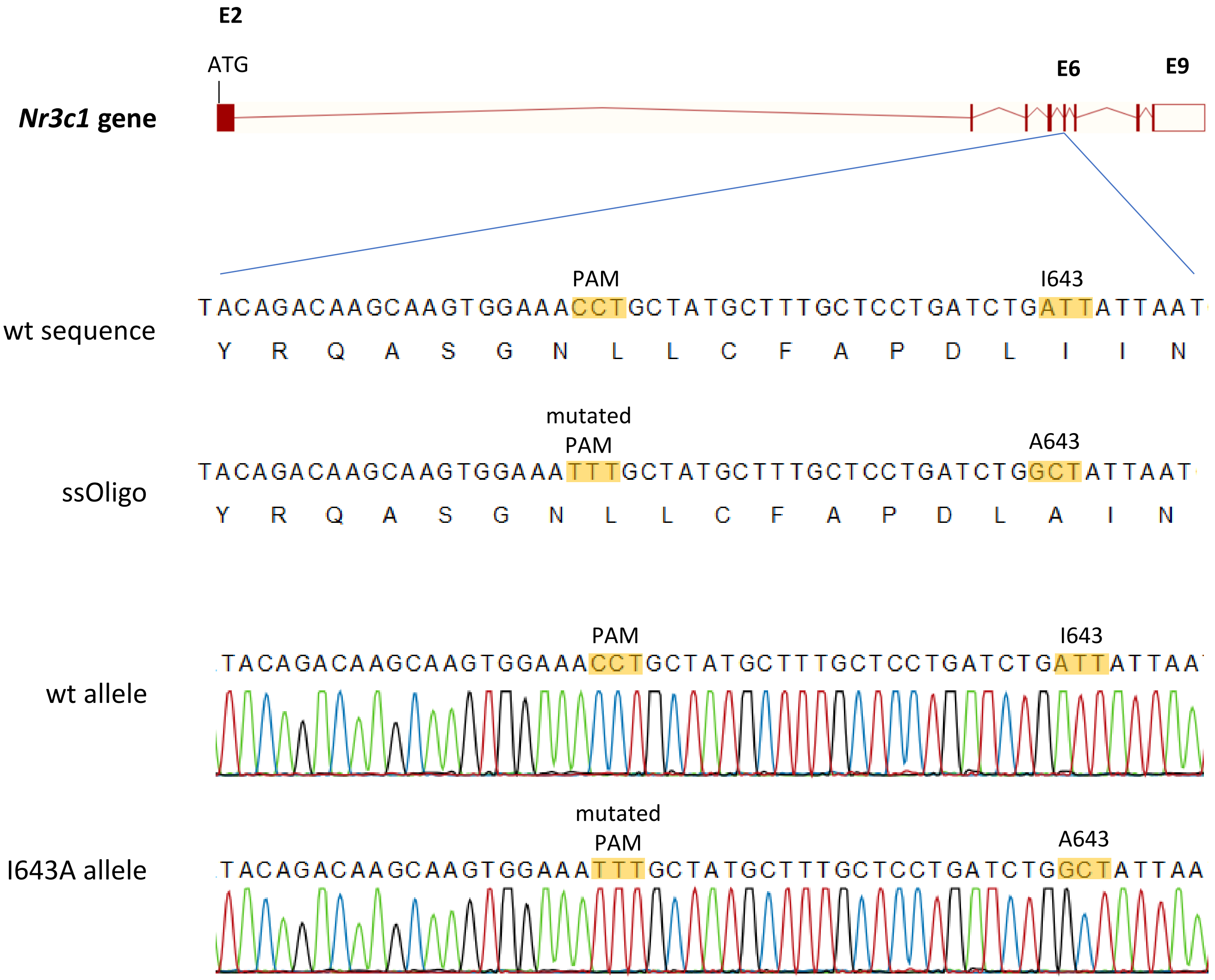

CRISPR/Cas mutagenesis strategy to mutate the Isoleucin on position 634 to an Alanine by mutating the codon. The general genomic structure of the *Nr3c1* locus is depicted. Part of the sequence of the sixth exon is shown as 'wt sequence'. The single stranded oligo, used in combination with the single guide RNA (sgRNA)is shown. It will mutate the relevant exon and mutate the PAM sequence, preventing further cutting by the sg RNA. Sequences obtained from wt mice and mutated mice, are shown.

Supplemental Figure 2

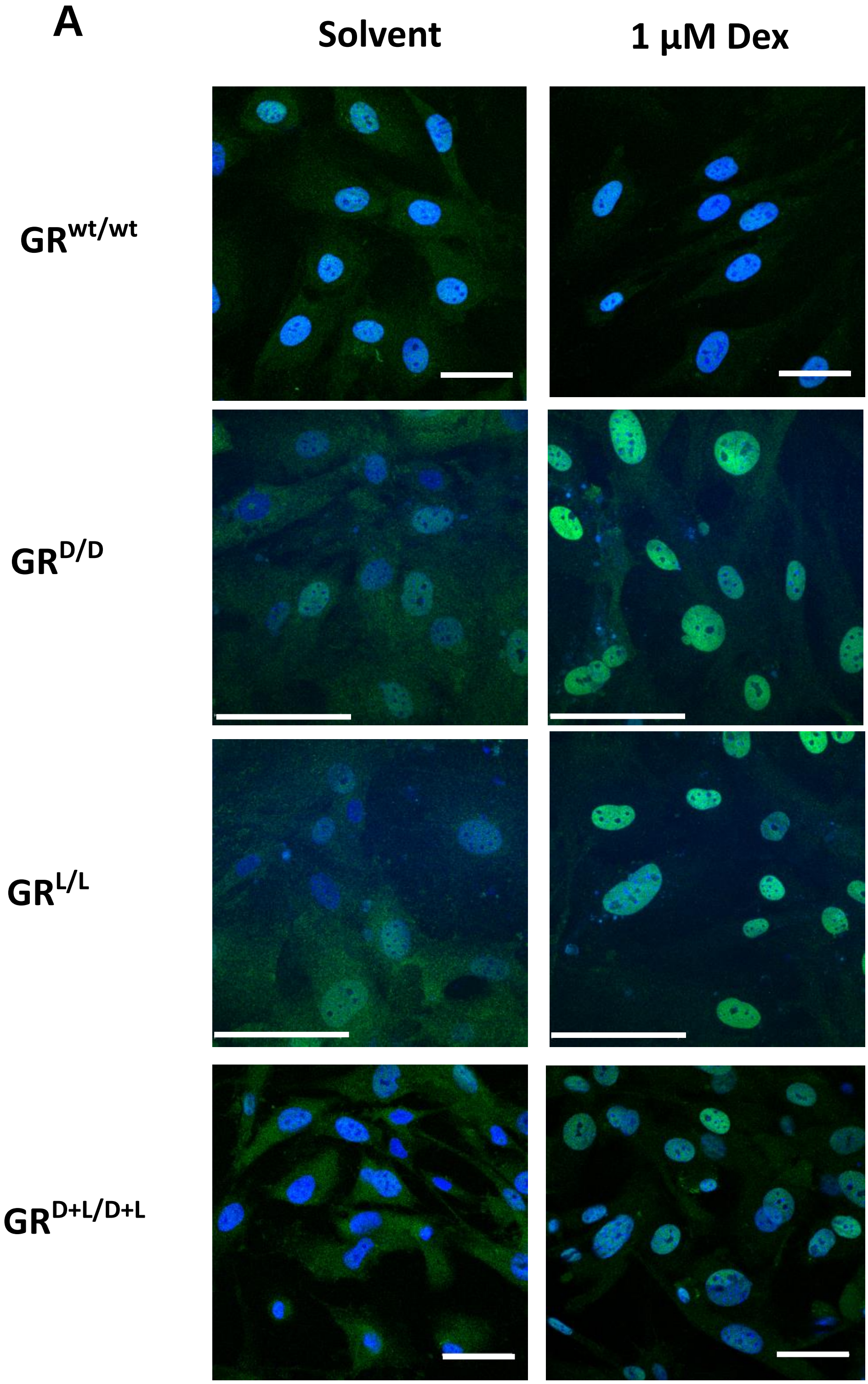

Nuclear translocation of GR in GR$^{wt/wt}$ and GR$^{D/D}$, GR$^{L/L}$ and GR$^{D+L/D+L}$ MEF cells, 15 minutes after stimulation with 1 µM DEX or with solvent (0,01% ethanol). GR is stained in green and nuclei in blue. Scale bars are 50 µm.

Supplemental Figure 2 cont.

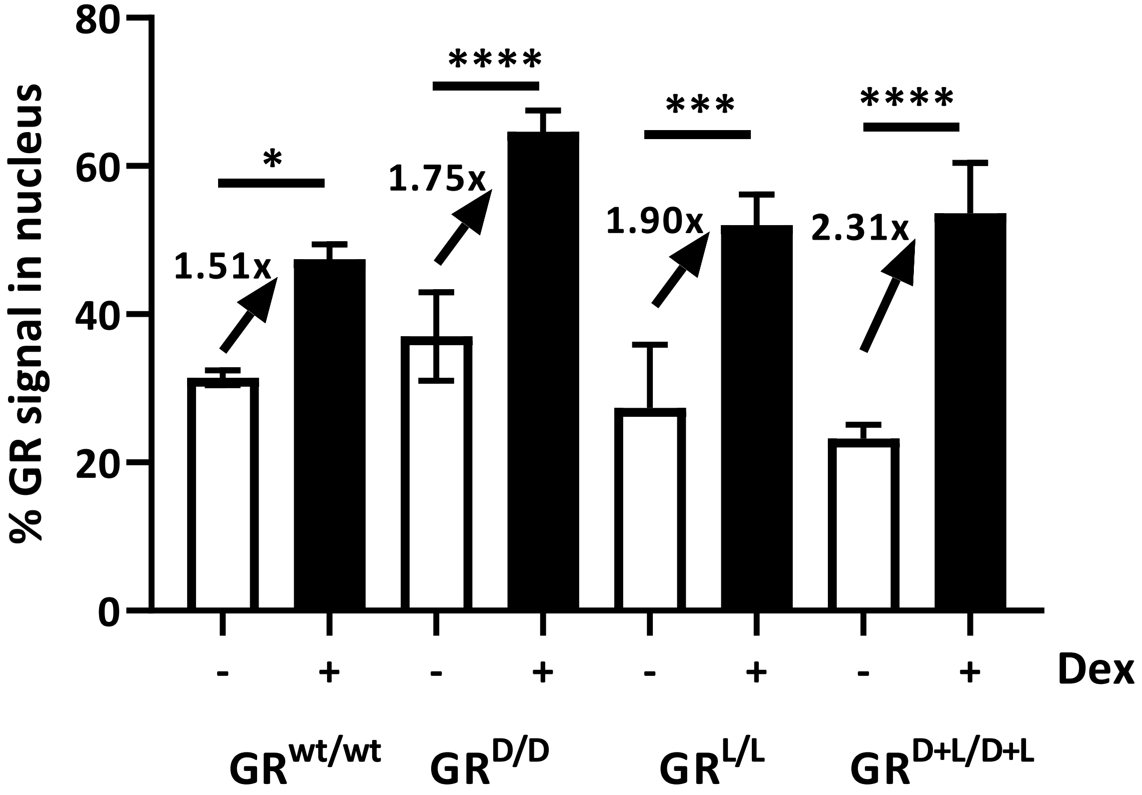

Images were taken from primary MEF cells derived from 3 mice per genotype to obtain biological replicates and stimulated with 1 µM Dex. A total of 4 Z-stacks per sample were imaged to obtain sufficient imaged cells for quantitative analysis. The plot shows the proportion of nuclear GR signal compared to total GR signal in the images. N=3 in each condition. Signal intensities were compared with a two-way ANOVA test using GraphPad Prism and post-hoc tests. Fold effects by Dex are mentioned in the graph.

Supplemental Figure 3

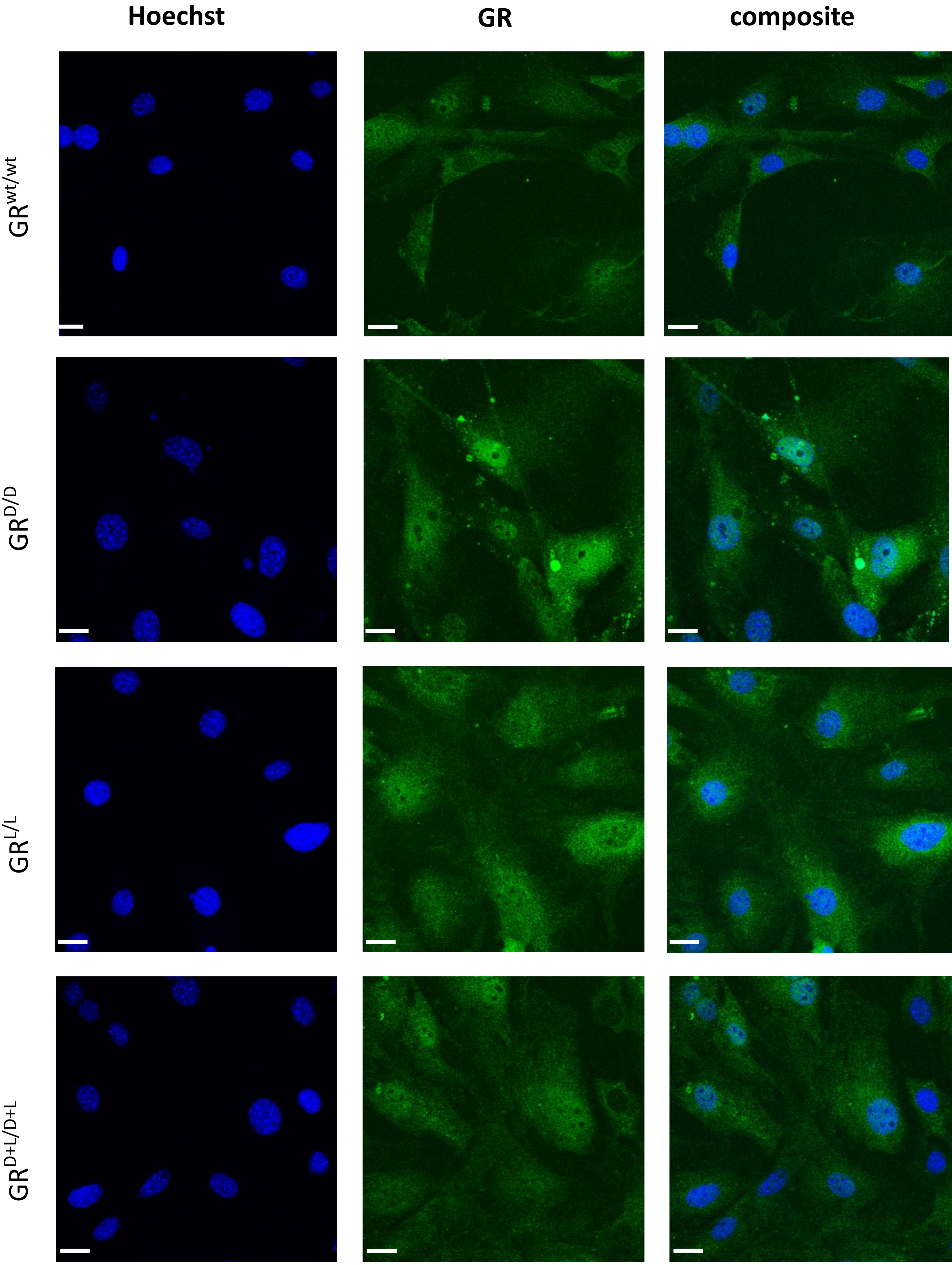

Supplemental Figure 3 cont.

Nuclear translocation with solvent in all GR genotypes. This shows the individual channel images from the 0 nM DEX (vehicle control) samples shown in Figure 5C. Scale bars are 22 µm.

Supplemental Figure 4

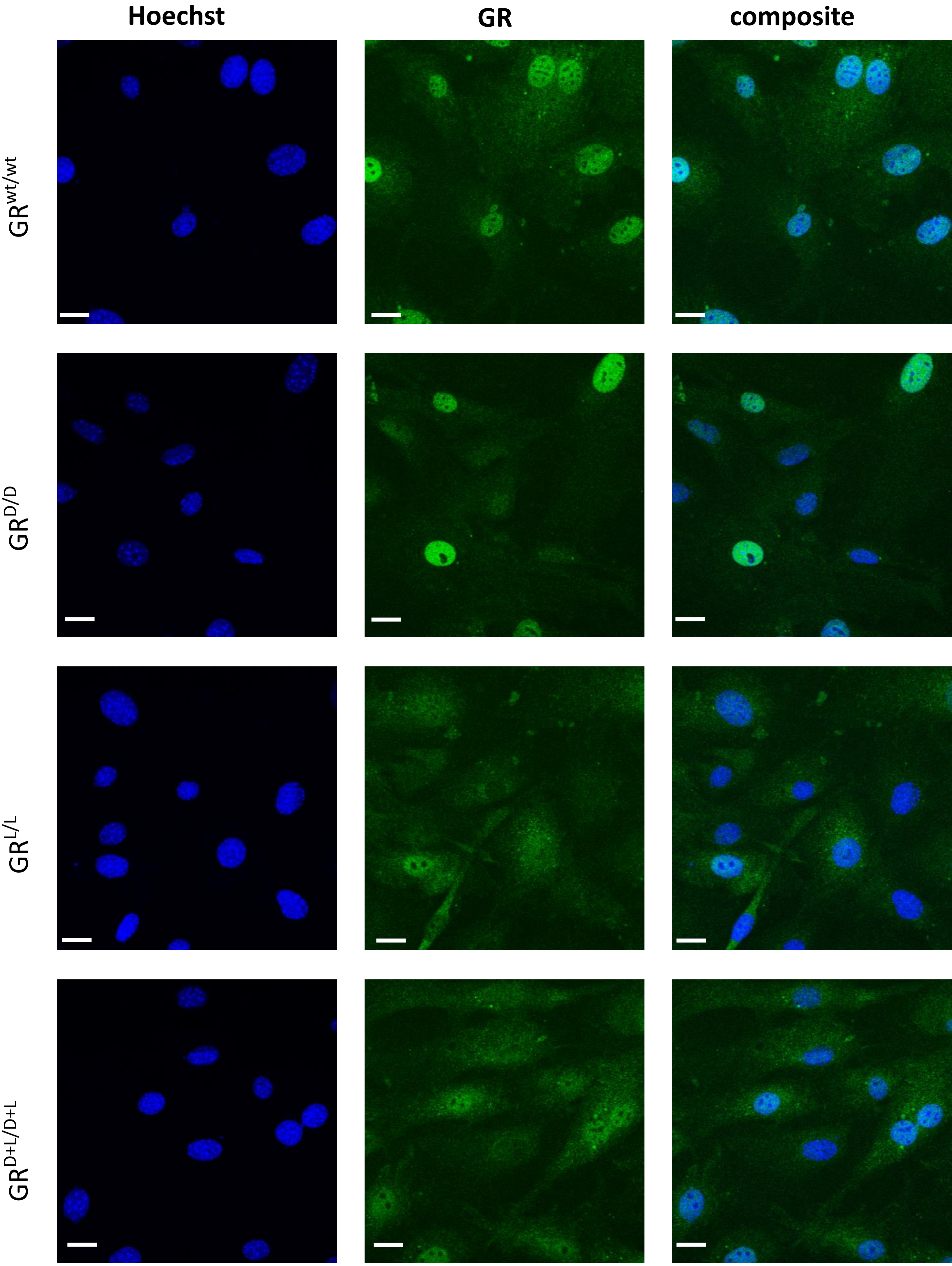

Supplemental Figure 4 cont.

Nuclear translocation with 10 nM DEX in all GR genotypes. This shows the individual channel images from the 10 nM DEX-stimulated samples shown in Figure 5C. Scale bars are 22 µm.

Supplemental Figure 5.

```
SP|P49843|GCR_ONCMY              LHLDDQMTLLQCSWLFLMSFGLGWRSYQQCNGGMLCFAPDLVINDERMKLPYMTDQCEQM 621
SP|Q5R9P5|GCR_PONAB              LHLDDQMTLLQYSWMFLMAFALGWRSYRQSSANLLCFAPDLIINEQRMTLPCMYDQCKHM 646
SP|P59667|GCR_RABIT              LHLDDQMTLLQYSWMFLMAFALGWRSYKQSSGNMLCFAPDLVINEQRMTLPYMYDQCKHM 641
SP|Q6XLJ0|GCR_CALJA              LHLDDQMTLLQYSWMFLMAFALGWRSYRQASSNLLCFAPDLIINEQRMTLPCMYDQCKHM 646
SP|O13186|GCR_SAIBB              LHLDDQMTLLQYSWMFLMAFALGWRSYRQASSNLLCFAPDLIINEQRMTLPCMYDQCKHM 646
SP|P06536|GCR_RAT                LHLDDQMTLLQYSWMFLMAFALGWRSYRQSSGNLLCFAPDLIINEQRMSLPCMYDQCKHM 664
SP|Q9N1U3|GCR_PIG                LHLDDQMTLLQYSWMFLMVFALGWRSYRQSSASLLCFAPDLVINEQRMALPCMYDQCRHM 651
SP|P06537|GCR_MOUSE              LHLDDQMTLLQYSWMFLMAFALGWRSYRQASGNLLCFAPDLIINEQRMTLPCMYDQCKHM 652
SP|P04150|GCR_HUMAN              LHLDDQMTLLQYSWMFLMAFALGWRSYRQSSANLLCFAPDLIINEQRMTLPCMYDQCKHM 646
SP|O46567|GCR_SAISC              LHLDDQMTLLQYSWMFLMAFALGWRSYRQASSNLLCFAPDLIINEQRMTLPCMYDQCKHM 647
SP|P79269|GCR_SAGOE              LHLDDQMTLLQYSWMFLMAFALGWRSYRQASSNLLCFAPDLIINEQRMTLPCMYDQCKHM 646
SP|P49115|GCR_CAVPO              LHLDDQMTLLQYSWMFLMAFALGWRSYKQSNGSLLCFAPDLIINEQRMSLPWMYDQCRYM 640
SP|P79686|GCR_AOTNA              LHLDDQMTLLQYSWMFLMAFALGWRSYRQASSNLLCFAPDLIINEQRMTLPCMYDQCKHM 646
SP|Q95267|GCR_TUPBE              LHLDDQMTLLQYSWMFLMAFALGWRSYKQASANLLCFAPDLIINEQRMSLPFMYDQCKHM 645
TR|Q1XHK0|Q1XHK0_DANRE           LHLDDQMTLLQCSWLFIMSFGLGWRSYQHCNGNMLCFAPDLVINEERMKLPYMSDQCEQM 615
SP|P49844|GCR_XENLA              LHLDDQMTLLQYSWMFLMVFALGWRSYKQTNGSILYFAPDLVITEDRMHLPFMQERCQEM 645
SP|O73673|GCR_PAROL              LHLDDQMTLLQCSWLFLMSFSLGWRSYEQCNGNMLCFAPDLVINKERMKLPFMTDQCEQM 676
TR|A0A1D5PRD7|A0A1D5PRD7_CHICK LHLDDQMTLLQYSWMFLMAFALGWRSYKQSNGNLLCFAPDLIINEQRMNLPCMYEQCKHM 641
TR|H2QRP6|H2QRP6_PANTR           LHLDDQMTLLQYSWMFLMAFALGWRSYRQSSANLLCFAPDLIINEQRMTLPCMYDQCKHM 647
TR|F1Q298|F1Q298_CANLF           LHLDDQMTLLQYSWMFLMAFALGWRSYRQSSGSMLCFAPDLIINEQRMTLPCMYDQCKHM 650
TR|F1MN19|F1MN19_BOVIN           LHLDDQMTLLQYSWMFLMAFALGWRSYRQSCANMLCFAPDLIINEQRMALPCMYDQCKHM 650
TR|A0A2I2YDJ3|A0A2I2YDJ3_GORGO LHLDDQMTLLQYSWMFLMAFALGWRSYRQSSANLLCFAPDLIINEQRMTLPCMYDQCKHM 647
TR|H9FTF1|H9FTF1_MACMU           LHLDDQMTLLQYSWMFLMAFALGWRSYRQSSANLLCFAPDLIINEQRMTLPCMYDQCKHM 646
TR|G1NAS4|G1NAS4_MELGA           LHLDDQMTLLQYSWMFLMAFALGWRSYKQSNGNLLCFAPDLIINEQRMNLPCMYEQCKHM 640
TR|G1KP59|G1KP59_ANOCA           LHLDDQMTLLQYSWMFLMAFALGWRSYKQSNGSLLCFAPDLIINEQRMNLPCMYEQCKRL 642
TR|K7GAE8|K7GAE8_PELSI           LHLDDQMTLLQYSWMFLMAFALGWRSYRQSNGNLLCFAPDLIINEQRMNLPCMYDQCKHM 636
TR|G1PHX2|G1PHX2_MYOLU           LHLDDQMTLLQYSWMFLMAFALGWRSYRQSNANMLCFAPDLVINEQRMSLPFMYDQCKHM 645
TR|M3Y916|M3Y916_MUSPF           LHLDDQMTLLQYSWMFLMAFALGWRSYRQSSGNVLCFAPDLIINEQRMSLPCMYEQCKHM 651
TR|F6W567|F6W567_ORNAN           LHLDDQMTLLQYSWMFLMAFALGWRSYKQSSANLLCFAPDLIINEQRMNLPCMYDQCRHM 644
TR|M3ZBD8|M3ZBD8_NOMLE           LHLDDQMTLLQYSWMFLMAFALGWRSYRQSSANLLCFAPDLIINEQRMTLPCMYDQCKHM 647
TR|A0A2R9BT94|A0A2R9BT94_PANPA LHLDDQMTLLQYSWMFLMAFALGWRSYRQSSANLLCFAPDLIINEQRMTLPCMYDQCKHM 646
TR|H0VUL7|H0VUL7_CAVPO           LHLDDQMTLLQYSWMFLMAFALGWRSYKQSNGSLLCFAPDLIINEQRMSLPWMYDQCRYM 641
TR|W5NVF6|W5NVF6_SHEEP           LHLDDQMTLLQYSWMFLMAFALGWRSYRQSSANMLCFAPDLVINEQRMALPCMYDQCKHM 650
TR|A0A452EXA0|A0A452EXA0_CAPHI LHLDDQMTLLQYSWMFLMAFALGWRSYRQSSANMLCFAPDLVINEQRMALPCMYDQCKHM 650
TR|A0A452HUM1|A0A452HUM1_9SAUR LHLDDQMTLLQYSWMFLMAFALGWRSYKQSNGNLLCFAPDLIINEQRMNLPCMYDQCKHM 636
TR|A0A151M6S0|A0A151M6S0_ALLMI LHLDDQMTLLQYSWMFLMSFALGWRSYKQSNGNLLCFAPDLIINEQRMSLPCMYEQCKNM 611
TR|A0A4W4EHL9|A0A4W4EHL9_ELEEL LHLDDQMTLLQCSWLFLMSFSLGWRSYQQCNAGMLCFAPDLVINEERMKLPYMGEQCRQM 612
TR|A0A3P9IVA5|A0A3P9IVA5_ORYLA LHIDDQMTLLQCSWLFLMSFSLGWRSYEQCNGSMLCFAPDLVINTDRMQLPFMTDQCDQM 623
TR|A0A2D0SZD0|A0A2D0SZD0_ICTPU LHLDDQMTLLQCSWLFLMSFSLGWRSYQQCNGGMLCFAPDLVINEERMKLPYMSEQCEQM 568
TR|A0A3Q3SWH1|A0A3Q3SWH1_9TELE LHLDDQMTLLQCSWLFLMSFSLGWRSYEQCNGSMLCFAPDLVINKERMKLPFMTDQCQQM 663
TR|A0A673WSI9|A0A673WSI9_SALTR LHLDDQMTLLQCSWLFLMSFGLGWRSYQQCNGGMLCFAPDLVINDERMKLPYMTDQCEQM 615
```

Sequence alignment of part of the GR LBD of 41 (informative) eukaryotic species. Of the six amino acids involved in this LBD dimerization interphase, only the I634 is tolerated to mutate, albeit only to a valine, in some species. The P, involved in the hydrophobic interaction with the I634 of the other monomer is emphasized by a red box, as well as the I/V.

Supplemental Figure 6

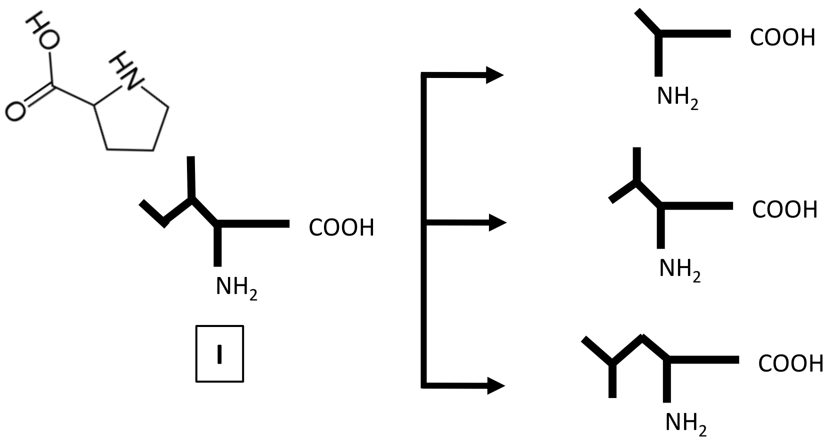

	PROVEAN	SIFT
A	-3,39 Deleterious	0 Damaging
V	+0,31 Neutral	1 Tolerated
L	-1,85 Neutral	0,002 Damaging

Prediction by PROVEAN and SIFT that replacement of I634 to a V634 is neutral and tolerated, but not a replacement to an A634 or L634.

Supplemental Figure 7

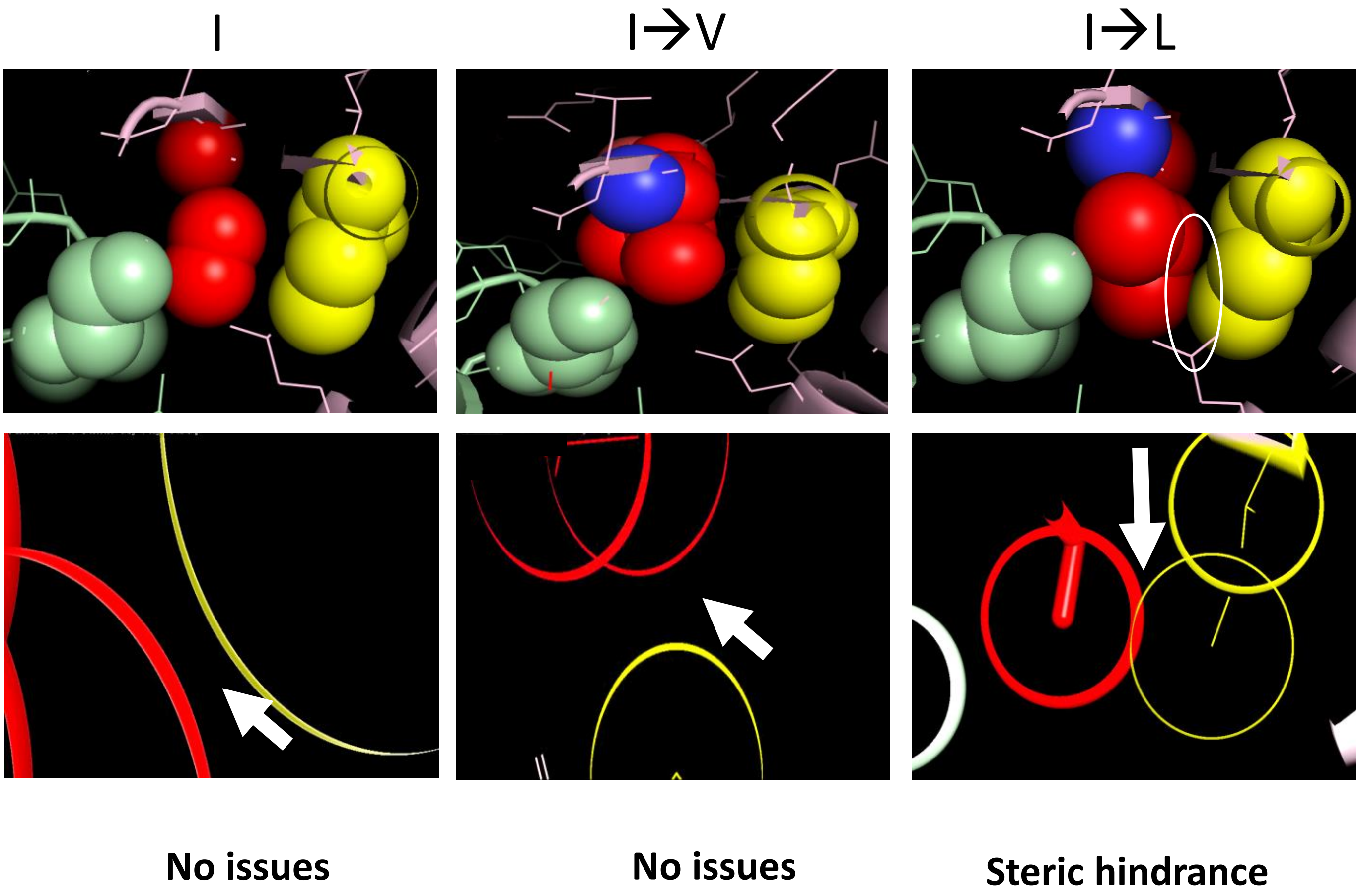

Explanation of the tolerance of Valine and Intolerance of Leucine at the LBD dimerization interphase. In red is position 628 (human), yellow position 622 (human) and light green position 625’ (human) of a second GR residue. In normal GR, the I628 interacts with P625’. An I→V allows stabilizing interaction between V628 and P625’. However, an I→L allows the interaction with P625’, but will cause steric hinderance with C622, preventing proper formation of the dimerization interface. The lower panels show the close distance between the atoms of the 628 residue and C622 and the incompatibility of a L628 (emphasized by the white arrows in the middle of the figures).

Supplemental Figure 8

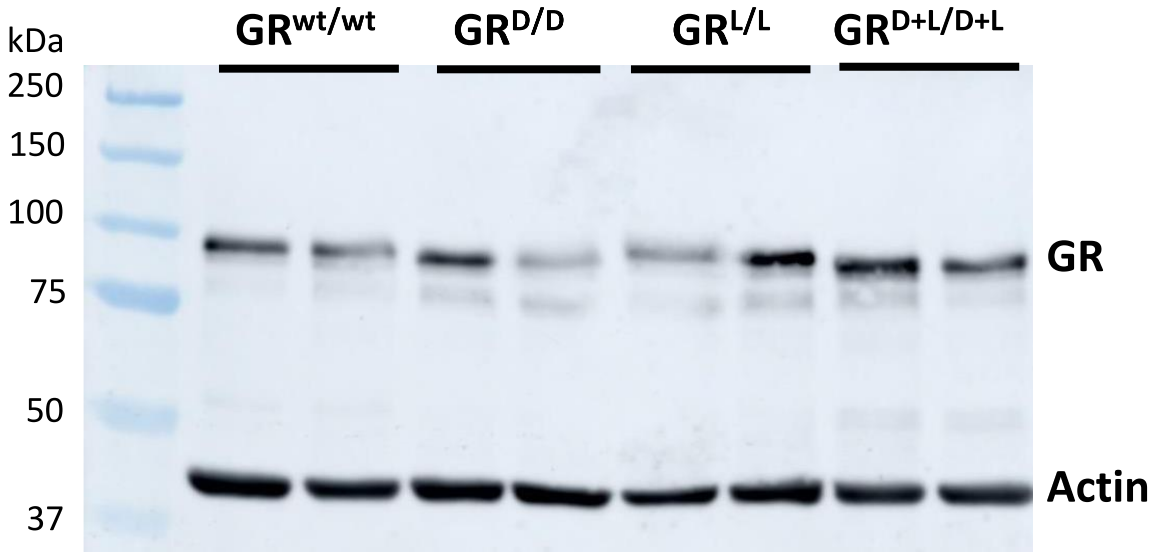

Western blot of GR quantification in all genotypes expressing mutant GR (MEF cells)
