# Supplementary material for: Point mutation I634A in the glucocorticoid receptor causes embryonic lethality by reduced ligand binding
Source: J Biol Chem. 2022 Jan 8;298(2):101574. doi: 10.1016/j.jbc.2022.101574 (PMC8808175; doi:10.1016/j.jbc.2022.101574)
Supplement: Supplemental Table S1 [file mmc2.docx]

**Supplemental Table 1: qPCR primers applied in this study**

| Gene | Forward primer | Reverse primer |
| --- | --- | --- |
| *Tsc22d3* | CCAGTGTGCTCCAGAAAGTGTAAG | AGAAGGCTCATTTGGCTCAATCTC |
| *Fkbp5* | TGAGGGCACCAGTAACAATGG | CAACATCCCTTTGTAGTGGACAT |
| *Dusp1* | GTTGTTGGATTGTCGCTCCTT | TTGGGCACGATATGCTCCAG |
| *Rpl* | CCTGCTGCTCTCAAGGTT | TGGTTGTCACTGCCTCGTACTT |
| *Ubc* | AGGTCAAACAGGAAGACAGACGTA | TCACACCCAAGAACAAGCACA |
