# Supplementary material for: Point mutation I634A in the glucocorticoid receptor causes embryonic lethality by reduced ligand binding
Source: J Biol Chem. 2022 Jan 8;298(2):101574. doi: 10.1016/j.jbc.2022.101574 (PMC8808175; doi:10.1016/j.jbc.2022.101574)
Supplement: Supplemental Table S2 [file mmc3.docx]

|  | **GR^+/+^** | **GR^D/D^** | **GR^L/L^** | **GR^D+L/D+L^** | **GR^-/-^** |
| --- | --- | --- | --- | --- | --- |
|  | | | | | |
| **Viability** | Y | Y | N | N | N |
| **Lung maturation** | Y | Y | N | N | N |
| **Skin stratification** | Y | Y | N | N | N |
|  | | | | | |
| **10^-6^ M Dex** | | | | | |
| Nuclear translocation | ++++ | ++++ | ++++ | ++++ | ND |
| Gene regulation | ++++ | ++ | +++ | + | 0 |
| Dimerization | ++++ | 0 | + | 0 | ND |
|  | | | | | |
| **10^-8^ M Dex** | | | | | |
| Nuclear translocation | ++++ | ++++ | 0 | 0 | ND |
| Gene regulation | ++++ | + | 0 | + | ND |
|  | | | | | |
| **Ligand binding (M)** | **3.3x10^-9^** | **2.4x10^-9^** | **4.5x10^-6^** | **7.5x10^-9^** | ND |

**Supplemental Table 2**: Overview of data obtained in this study. ND is not done.

|  |
| --- |
